# Plant microbiome analysis after *Metarhizium* amendment reveals increases in abundance of plant growth-promoting organisms and maintenance of disease-suppressive soil

**DOI:** 10.1371/journal.pone.0231150

**Published:** 2020-04-10

**Authors:** Larissa Barelli, Alison S. Waller, Scott W. Behie, Michael J. Bidochka

**Affiliations:** 1 Department of Biotechnology, Brock University, St. Catharines, ON, Canada; 2 Department of Biological Sciences, Brock University, St. Catharines, ON, Canada; 3 Department of Plant and Microbial Biology, University of California, Berkeley, CA, United States of America; Banaras Hindu University, INDIA

## Abstract

The microbial community in the plant rhizosphere is vital to plant productivity and disease resistance. Alterations in the composition and diversity of species within this community could be detrimental if microbes suppressing the activity of pathogens are removed. Species of the insect-pathogenic fungus, *Metarhizium*, commonly employed as biological control agents against crop pests, have recently been identified as plant root colonizers and provide a variety of benefits (e.g. growth promotion, drought resistance, nitrogen acquisition). However, the impact of *Metarhizium* amendment on the rhizosphere microbiome has yet to be elucidated. Using Illumina sequencing, we examined the community profiles (bacteria and fungi) of common bean (*Phaseolus vulgaris*) rhizosphere (loose soil and plant root) after amendment with *M*. *robertsii* conidia, in the presence and absence of an insect host. Although alpha diversity was not significantly affected overall, there were numerous examples of plant growth-promoting organisms that significantly increased with *Metarhizium* amendment (*Bradyrhizobium*, *Flavobacterium*, *Chaetomium*, *Trichoderma*). Specifically, the abundance of *Bradyrhizobium*, a group of nitrogen-fixing bacteria, was confirmed to be increased using a qPCR assay with genus-specific primers. In addition, the ability of the microbiome to suppress the activity of a known bean root pathogen was assessed. The development of disease symptoms after application with *Fusarium solani* f. sp. *phaseoli* was visible in the hypocotyl and upper root of plants grown in sterilized soil but was suppressed during growth in microbiome soil and soil treated with *M*. *robertsii*. Successful amendment of agricultural soils with biocontrol agents such as *Metarhizium* necessitates a comprehensive understanding of the effects on the diversity of the rhizosphere microbiome. Such research is fundamentally important towards sustainable agricultural practices to improve overall plant health and productivity.

## Introduction

Current agricultural practices are often damaging to the surrounding environment and require large energy inputs in the forms of fertilizers and pest control agents [1]. Therefore, in order to establish increasingly sustainable agricultural practices, the development of techniques designed to decrease these energy inputs is of immediate importance. One way to facilitate these goals would be through the increased integration of beneficial plant microbiomes (i.e. those enhancing plant growth, nutrient use efficiency, abiotic stress tolerance, and disease resistance) in the rhizosphere.

Agricultural plants utilize the complex community of rhizospheric microbes to maintain health and primary production [2]. These microbes vary in their ecological roles, and provide benefits that include the stimulation of plant growth [3], competitive suppression of pathogens through secondary metabolites or spatial restriction [4], increased resistance to biotic and abiotic stress by induced systemic resistance [5], and solubilization and transport of nutrients that would otherwise be unavailable to the plant [2,6].

The diversity of the rhizospheric community gives rise to a complex web of microbial interactions. This suggests a paucity of information regarding the cause of the observed plant phenotype relative to the current microbial community. There are numerous studies where microbial amendments have been applied to the rhizosphere in benefit of the plant. These include rhizobia inoculants for increasing nitrogen fixation [7,8], *Azospirillum* treatments that are phytostimulatory [9,10], mycorrhizal inoculants that deliver nutrients to the host plant [11–13] and biocontrol inoculants such as *Trichoderma harzianum* that demonstrate antagonism against fungal diseases [14–16]. However, the extent to which these inoculants affect the microbial (bacteria and fungi) rhizospheric community is influenced by numerous factors and remains an area of great interest and in need of research. Major questions remain regarding how the impact of microbial taxonomic groups can be related to the functional capabilities of the root microbiome.

*Metarhizium* is a genus of rhizosphere inhabiting, insect-pathogenic fungi that are ubiquitous in soil and currently used as a biocontrol agents against crop pests through crop or seed treatment with conidial formulations [17]. *Metarhizium* also provides benefits to a variety of host plants including resistance to salt stress [18], increased plant biomass and growth [18,19], stimulation of root growth [17,20], acquisition of insect-derived nitrogen [21,22], and antagonism of plant pathogens [23]. The plant growth-promoting qualities of *Metarhizium* coupled with its ability to parasitize insects make it an attractive candidate as a rhizospheric inoculant.

Here we grew common bean plants, *Phaseolus vulgaris*, in unsterilized *Metarhizium* amended soil, under laboratory conditions. As the plant associating lifestyle of *Metarhizium* may converge with the insect pathogenic lifestyle to differentially affect the microbiome, such as when insect-derived nitrogen is available for exchange with the plant [21], we also included *Galleria mellonella* larvae as a treatment. The bacterial and fungal community profiles from both rhizospheric soil and the plant root (rhizoplane and endosphere) were analyzed by Illumina sequencing. A generalized linear model was used to assess whether *Metarhizium* amendment had a significant effect. A quantitative PCR was performed to confirm the results of Illumina sequencing for the bacterial genus, *Bradyrhizobium*; identified as being significantly affected by *Metarhizium* application. To determine whether the described community had the capacity to suppress disease, plants were challenged with the known bean pathogen, *Fusarium solani* f. sp. *phaseoli*. An interactive website was created (https://metarhiz-microbiome.shinyapps.io/shiny3/) that allows for user-directed data analysis. This research is a necessary step in understanding the influence that amendment with *Metarhizium* has on the rhizospheric community under controlled conditions and could help elucidate other potential means of plant growth promotion through secondary interactions with microorganisms within the rhizospheric community.

## Materials and methods

### Biological materials

Haricot bean seeds (*Phaseolus vulgaris*; “soldier” variety) were purchased from OSC seeds (Ontario, Canada). The fungal lab strain *Metarhizium robertsii* (ARSEF 2575-GFP) that expresses green fluorescent protein (GFP), and *Fusarium solani* f. sp. *phaseoli*, were grown on potato dextrose agar (PDA) for 14 days to obtain conidia. The transformation of the *Metarhizium* strain has been previously described [24]. Conidia were harvested with 0.01% Triton X-100 and the suspensions were adjusted to a final concentration of 1.5 x 10^6^ conidia/mL. Wax moth larvae (*Galleria mellonella*) were purchased from a local bait shop (Missassauga Imports). Unsterilized soil (clay:loam mix) collected from a farm (Pelham, Ontario) in April before any crop had been planted and was sifted to remove large debris and used directly in the experiment. The farm grew corn the previous year and before that the land was fallow for at least 10 years.

### Experimental setup

Bean seeds were surface sterilized by immersion in 2% NaOCl for 5 minutes, rinsed with sterile water and then immersed in 15% H_2_O_2_ for 20 minutes. Seeds were rinsed a minimum of three times with sterile water until no peroxide remained. A 100uL aliquot of the last water rinse was plated on PDA to ensure sterility. Bean seeds were germinated on water agar and individually placed in 9-cm plastic pots filled with field-collected soil, for a total of 36 potted plants (S1 Table). To eighteen pots, 3 larvae each were buried in the soil below the seedling and of these pots, nine had 5 mL of a 1.5 x 10^6^ conidia/mL suspension of *M*. *robertsii* 2575-GFP added to the soil surface, and nine received only water. To the remaining eighteen pots without larvae, nine pots were amended with 5mL conidial suspension, and nine pots received only water. A final nine pots were filled with only the field-collected soil (“bulk soil”; no plant, no treatment). Plants were incubated at 25°C with 50–60% humidity, and under a 16/8-hour light/dark cycle for 14 days.

### Plant harvest and DNA extraction

Rhizospheric soil was collected by removing bulk soil from the roots with gloved hands and gently shaking the root above a weigh dish until 1 g was obtained. The soil was sieved through a 1 mm^2^ mesh and 250 mg of soil was taken from each sample, including the pots without a bean plant, and processed as recommended by the manufacturer for extraction of DNA using the Soil Plus DNA mini kit (Norgen Biotek). The remaining root was washed gently under running tap water to remove residual soil and DNA was extracted from the entire root system using the CTAB protocol described below. Samples were pooled in groups of three for a total of 27 samples (15 soil, 12 root). DNA was quantified using Qubit 3.0^™^ (Thermo Fisher).

### CTAB DNA extraction

The fresh root tissue was weighed and then immediately flash frozen in liquid nitrogen and ground to fine powder using a mortar and pestle. A 2X CTAB solution [2% (w/v) hexadecyltrimethylammonium bromide (CTAB), 100 mM Tris (pH 8.0), 20 mM EDTA (pH 8.0), and 1.4 M NaCl was autoclaved sterile, to which 2% (w/v) of polyvinylpyrrlidone (M_w_ 40,000) and 1% 2-mercaptoethanol are added] was preheated to 65°C and applied to the root powder at a volume of 5:1 (i.e. 5 mL 2X CTAB per 1 g of fresh root weight) and vortexed for 1 minute. The tube was incubated in a 65°C water bath for 30 minutes. After incubation, the tube was vortexed again for 10 seconds to homogenize the solution, and then a 1 mL aliquot was transferred to a 2 mL tube. One volume of chloroform:isoamyl alcohol (24:1) was added to the supernatant and vigorously shaken to form an emulsion, followed by centrifugation at 14,000 rpm for 2 minutes. This step was repeated once. The supernatant was transferred to a 1.5 mL microfuge tube and 0.7 volumes of isopropanol were added and mixed by inversion to precipitate the DNA. The tube was centrifuged at 14,000 rpm for 5 minutes. The DNA pellet was washed once with 70% ethanol and once with 100% ethanol, being centrifuged at 14,000 rpm for 2 minutes each time. The ethanol was decanted, and the pellet allowed to dry for 10 minutes on the bench top. The pellet was resuspended in 50 μL of 1X TE buffer pH 8.0.

### CFU determination of fungal inoculum

To the remaining 0.75 g sieved soil of each sample, 5 mL of 0.01% Triton X-100 was added and the sample vortexed vigorously. A 100 uL aliquot was plated, in duplicate, on CTC [25] agar plates and incubated at 27°C for 5 days. Colonies of *M*. *robertsii* were verified by visualization of GFP fluorescence and counted.

### Next generation sequencing, sequence processing, and statistical analyses

DNA samples were sent to Microbiome Insights (Vancouver, Canada) for analysis. The 16S ribosomal RNA genes (V4 region) and ITS2 region genes were sequenced on an Illumina MiSeq (v. 2 chemistry) using the dual barcoding protocol [26]. Primers and PCR conditions used for 16S sequencing are identical to those of Kozich et al. [26]; those used for ITS2 sequencing were described by Gweon et al. [27]. Bacterial Raw Fastq files were quality-filtered and clustered into 97% similarity operational taxonomic units (OTUs) using the mothur software package [28]. Sequences were processed and clustered into operational taxonomic units (OTUs) with the mothur software package (v. 1.39.5)[28], following the recommended procedure (https://www.mothur.org/wiki/MiSeq_SOP; accessed Aug 2018). Paired-end reads were merged and curated to reduce sequencing error [29]. Chimeric sequences were identified and removed using VSEARCH [30]. The curated sequences were assigned to OTUs at 97% similarity using the OptiClust algorithm [31] and classified to the deepest taxonomic level that had 80% support using the naive Bayesian classifier trained on the Greengenes taxonomy outline (version 13.8)[32]. A total of 189,365 high-quality bacterial reads was obtained with a final 16S dataset of 16,356 OTUs (including those occurring once with a count of 1) and a read range of 2,442 and 12,606. High quality reads were classified using Greengenes (v. 13.8) [32] as the reference database. There were 914,945 high-quality fungal reads with a final ITS2 dataset containing 3,409 OTUs (including those occurring once with a count of 1) and a read range of 10,613 and 68,269. The processing pipeline was identical as the one used for bacteria, except for the following differences: paired-end reads were trimmed at the non-overlapping ends, and high quality reads were classified using UNITE (v. 7.1) [33] as the reference database. A consensus taxonomy for each OTU was obtained and the OTU abundances were then aggregated at multiple taxonomic levels (genus, family, phylum) (S1 File). Data analysis was performed using R statistical programming language [34]. OTU abundances were summarized with the Bray-Curtis index and a principle coordinate analysis (PCoA) was performed to visualize microbiome similarities and a permutational analysis of variance (PERMANOVA) was done to test the significance of groups. Alpha diversity was calculated using Shannon’s diversity index. For analysis of differential OTU abundances the R package ALDEX2 [35] was used in which technical variation is assessed by Monte-Carlo sampling from a Dirichlet distribution, returning a multivariate probability distribution, from which the centred log-ratio is then calculated. The development version was used as it allows for generalized linear models that test for interactions between two covariates. Welch’s t-tests with an adjusted P value were used for pairwise comparisons. Summarized statistical results are shown in supporting information S3 to S8 Tables. The interactive website (S2 File) allows for examination of the data and download of the R scripts, which is also provided as supporting information (S3 File). Raw sequences are available from the Short Read Archive (NCBI) (Accession: PRJNA558088).

### *Bradyrhizobium* qPCR confirmation and culturing

To confirm the results of Illumina sequencing that detected an increase in abundance of *Bradyrhizobium*, a real-time PCR was performed using the genus-specific primers 691-709fAP (5’-GTGAAATDCGTAGAKATT-3’) and 907r (5’-CCGTCAATTCMTTTRAGTTT-3’) [36]. A 20 μL qPCR reaction utilized the SensiFAST SYBR no-rox kit (Bioline), 0.125uM of each primer, and 10 ng of DNA from the original extracted samples. A standard curve was created from DNA extracted from a culture of *Bradyrhizobium japonicum* generously provided by Dr. Edward Topp (Agriculture and Agri-Food Canada). The qPCR protocol followed the standard manufacturer’s conditions and results were analyzed using CFX^™^ Manager version 3.1 (Bio-Rad). To ensure that the presence of *Bradyrhizobium* was not from DNA contamination [37] 100 μL aliquots of soil samples (described above in CFU determination of fungal inoculum) were plated on yeast-mannitol selective media [(g L^-1^): mannitol (10.0); K_2_HPO_4_ (0.25); KH_2_PO_4_ (0.35); yeast extract (1.7); MgSO_4_•7H_2_O, (0.15); NaCl (0.1); CaCl_2_•2H_2_O (0.08); Chloramphenicol (0.03); Tetracycline (0.01); Cycloheximide (0.05)] [38] and incubated at 27°C for 14 days. Colony PCR was performed on all colonies that appeared similar to the *B*. *japonicum* strain using the universal bacteria primers 27F/1492R in a standard PCR reaction (New England Biolabs) according to manufacturer instructions. The approximately 1500 nucleotide amplicon was sent for Sanger sequencing (TCAG, Sickkids, Toronto) and identified through BLASTn analysis.

### Microbiome disease-suppression activity assay

To determine the ability of the soil microbiome to suppress disease, the known bean root rot pathogen, *Fusarium solani* f. sp. *phaseoli* was applied via the soil-drench method to bean plants growing in autoclaved- or unautoclaved field-collected soil [39,40]. Small batches of the microbiome soil were autoclaved three times, with a period of 24 hours between cycles (121°C, 15 psi, 60 minutes). The soil-drench application consisted of a final volume of 5 mL of: *F*. *solani*, *F*. *solani* + *M*. *robertsii*, or water. A total of 3.75 x 10^6^ spores was applied for each fungus. Each condition had a total of three biological replicates. Co-inoculation of *F*. *solani* with *M*. *robertsii* was performed to confirm the ability of *M*. *robertsii* to antagonize *F*. *solani* [23]. Plants were incubated in the conditions stated above. After 14 days, plants were harvested and the roots were washed and examined for signs of root-rot disease such as discolouration of hypocotyl/upper tap root, lesion formation, and necrosis [41]. Two measures of disease severity were performed as previously described [23]. Root rot disease was scored from 0–1, with 0 indicating no root-rot symptoms and 1.0 indicating greater than 75% of rotted root area [42]. The disease index based on necrotic lesions was scored from 0–5, with 0 = no disease symptoms; 1 = slight browning, or <50% discolouration of the hypocotyl and firm upon pressure from thumb and forefinger, and slight root pruning; 2 = as 1 but >50% surface discolouration; 3 = discoloured hypocotyl and roots collapsing under pressure and extensive root pruning; 4 = darkly discoloured hypocotyl and roots completely collapsed or collapsing easily under pressure and severe root pruning; and 5 = dead or dying plant [43]. The difference between mean values for each disease index was evaluated with an ANOVA and Scheffe’s post-hoc (α = 0.05; n = 3).

### Data availability

To explore the data further please see the interactive website at: https://metarhiz-microbiome.shinyapps.io/shiny3/ (created by Dr. Alison S. Waller; S2 File). Here the trends between different treatments for bacterial and fungal OTUs of interest (at the genus, family and phylum level) can be examined. Alternatively, a factor of interest can be selected (e.g. *Metarhizium* addition, insect or sample type), and a P value and a list of OTUs that have significantly different abundances, based on generalized linear model (GLM) or distance-based Welch’s t-test, can be viewed. The raw data is available for download on this site as well as from the Short Read Archive of NCBI (Accession: PRJNA558088). Matrices of OTU abundance for bacteria and fungi at phylum, family, and genus level are available for download as well.

## Results

### *Metarhizium* inoculum is able to establish within native microbiota

**Fig 1:**
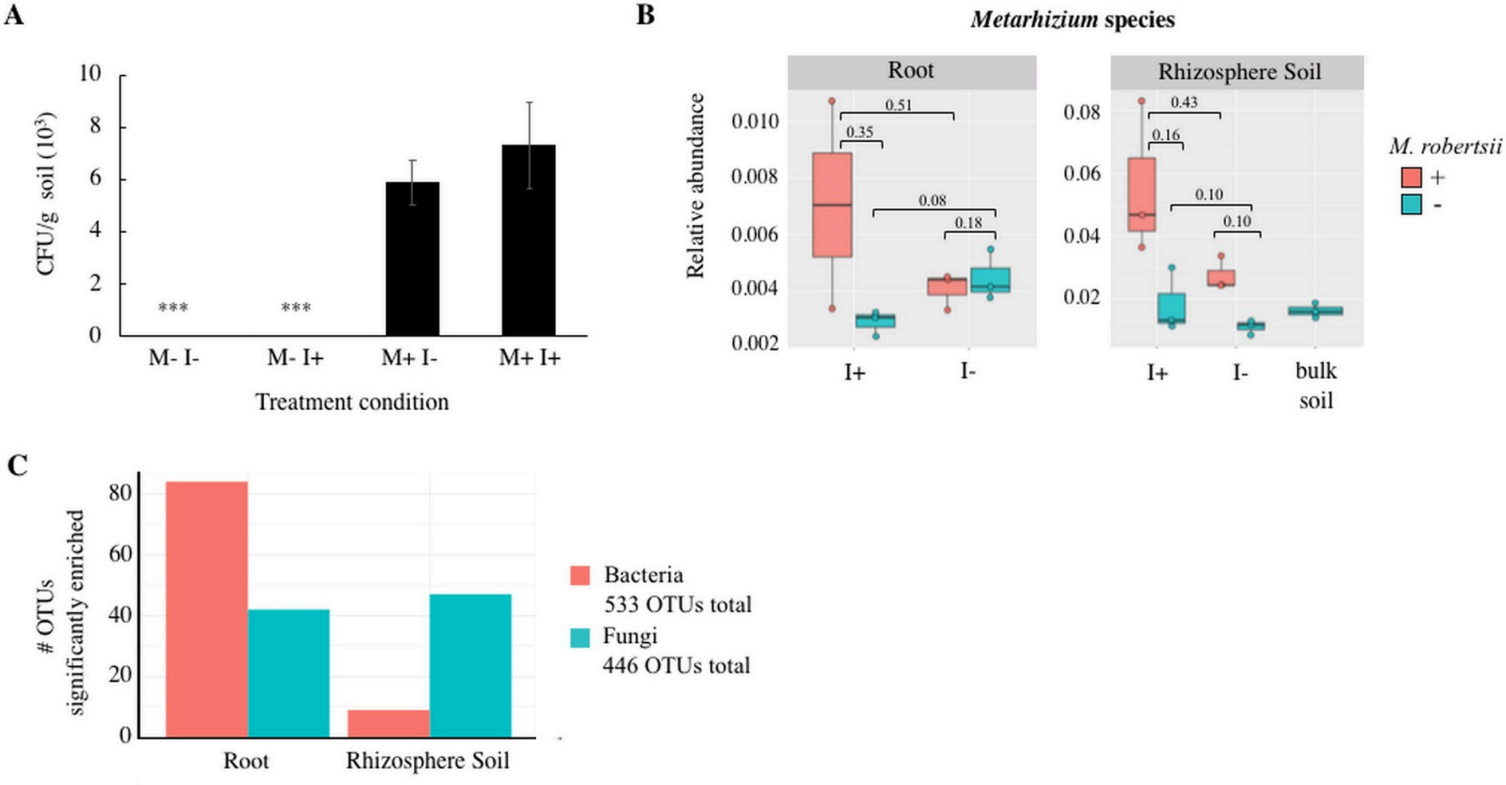
Determination of (A) CFU of *M*. *robertsii* 2575-GFP recovered from soil of potted bean plant treatments after 14 days, (B) relative abundance of *Metarhizium* species from Illumina sequencing, and (C) number of bacterial and fungal OTUs enriched in the root and rhizosphere soil compartments. Treatment conditions: M = *M*. *robertsii* 2575-GFP; I = Insect, *Galleria mellonella* larvae. Statistically significant differences in CFU g^-1^ soil of *M*. *robertsii* are shown by asterisks (ANOVA, F = 9.41, *P* < 0.001; n = 9). Enriched OTUs were determined from the generalized linear model (GLM).

As an internal control measure, colony forming units (CFU) g^-1^ soil of the fungal inoculum *M*. *robertsii* 2575-GFP was quantified, utilizing GFP fluorescence of colonies in order to ensure there was no cross contamination of sample treatments (Fig 1A). *M*. *robertsii* 2575-GFP was only detected in pots that received the conidial inoculum. This was necessary to confirm that any *Metarhizium* detected by Illumina sequencing in uninoculated samples was due to the natural occurrence of this fungus. Fig 1B shows the relative abundance of *Metarhizium* detected in all conditions as determined from Illumina sequencing. Overall, there was a higher abundance of *Metarhizium* detected in rhizospheric soil than with the root (rhizoplane and endosphere). Conidial inoculation resulted in a statistically significant increase in the abundance of *Metarhizium* when *G*. *mellonella* larvae were present (Welch’s t-test, *P* = 0.04).

Growth measurements determined that there were no significant differences in the dry weight of plants between any treatment (ANOVA, P = 0.39, n = 9) (Table 1).

**Table 1:** Dry weight measurements for bean plants.

	Dry weight (g)		
Treatment	Root	Phyllosphere	Total
Bean	0.11 ± 0.04	0.44 ± 0.14	0.54 ± 0.15
Bean + insect	0.11 ± 0.03	0.33 ± 0.11	0.44 ± 0.10
Bean + *Metarhizium*	0.11 ± 0.02	0.35 ± 0.09	0.46 ± 0.10
Bean + *Metarhizium* + insect	0.12 ± 0.02	0.34 ± 0.18	0.46 ± 0.17

Plants were treated with *Metarhizium robertsii* with and without insect larvae (*Galleria mellonela*) and grown in field-collected soil for 14 days. There was no significant difference between the conditions (ANOVA, *P* = 0.39; n = 9).

### Identification and diversity of bacteria in treatments

**Fig 2:**
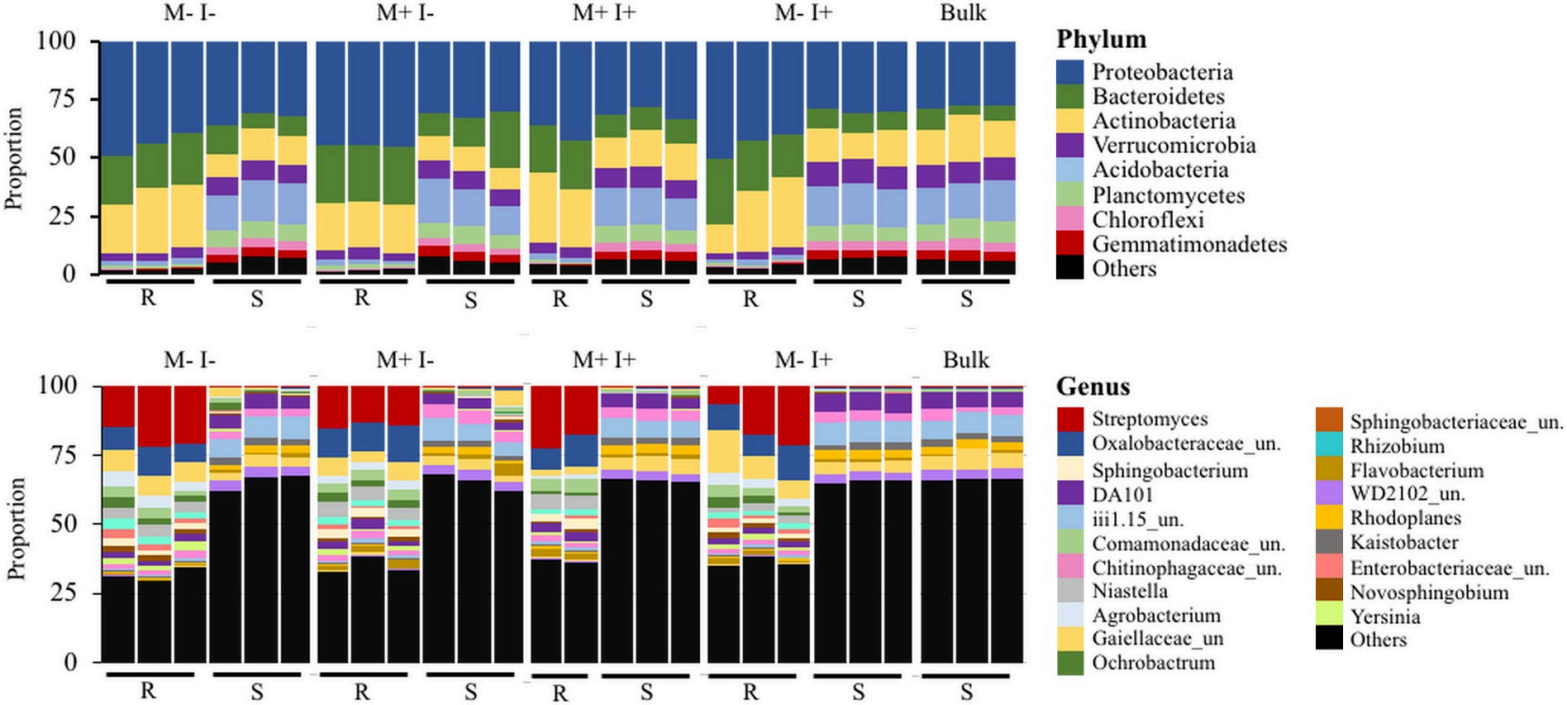
Proportional distribution of the major (abundance greater than 1%) bacterial phyla and genera present in *P*. *vulgaris* root (R) and rhizospheric soil (S). M: *M*. *robertsii* 2575-GFP. I: Insect, *Galleria mellonella*, larvae. Bulk soil represents original potting substrate (no bean plant). All conditions have three replicates which are pools of three samples each. “Others” signifies lower-abundance taxa. Un. = unclassified.

Through Illumina sequencing, a total of 533 bacterial OTUs were identified, with 84 and 9 enriched in the root and rhizosphere soil compartments, respectively (Fig 1C). The list of OTUs and the corresponding taxonomic classification can be viewed in S2 Table. The composition of bacterial phyla and the predominant genera (relative abundance above 1%) of the different treatments can be seen in Fig 2. In all treatments, the major phyla were Proteobacteria, Actinobacteria, and Bacteriodetes (Fig 2). However, there was a larger proportion of Acidobacteria and Verrucomicrobia in soil (~10–20%) compared to root (~2%). At the genus level, rare taxa (<1% abundance) accounted for nearly 70% of all taxa in soil, and approximately 35% in roots. Aside from the large proportion of lower-abundance taxa, *Streptomyces*, *Sphingobacterium* and unclassified *Oxalobacteraceae* and *Comamonadaceae* predominated in root samples (Fig 2). In contrast, DA101 and unclassified *Gaiellaceae*, *Chitinophagaceae*, and Acidobacteria (subdivision 6; iii1.15) were most abundant in soil samples.

**Fig 3:**
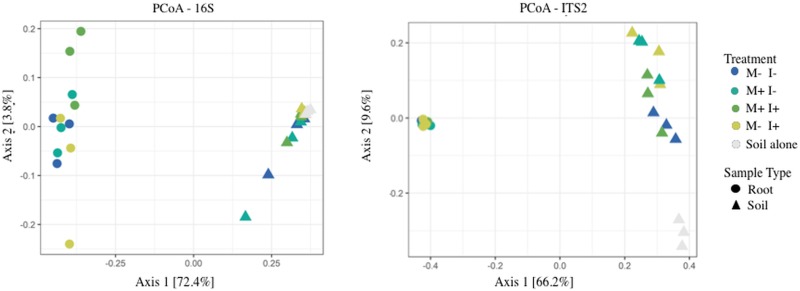
Principal coordinate analysis (PCoA) of bacterial and fungal genera from root and soil compartments. M: *M*. *robertsii* 2575-GFP. I: Insect, *Galleria mellonella*, larvae. Bulk soil represents original potting substrate (no bean plant). All conditions have three replicates which are pools of three samples each.

**Fig 4:**
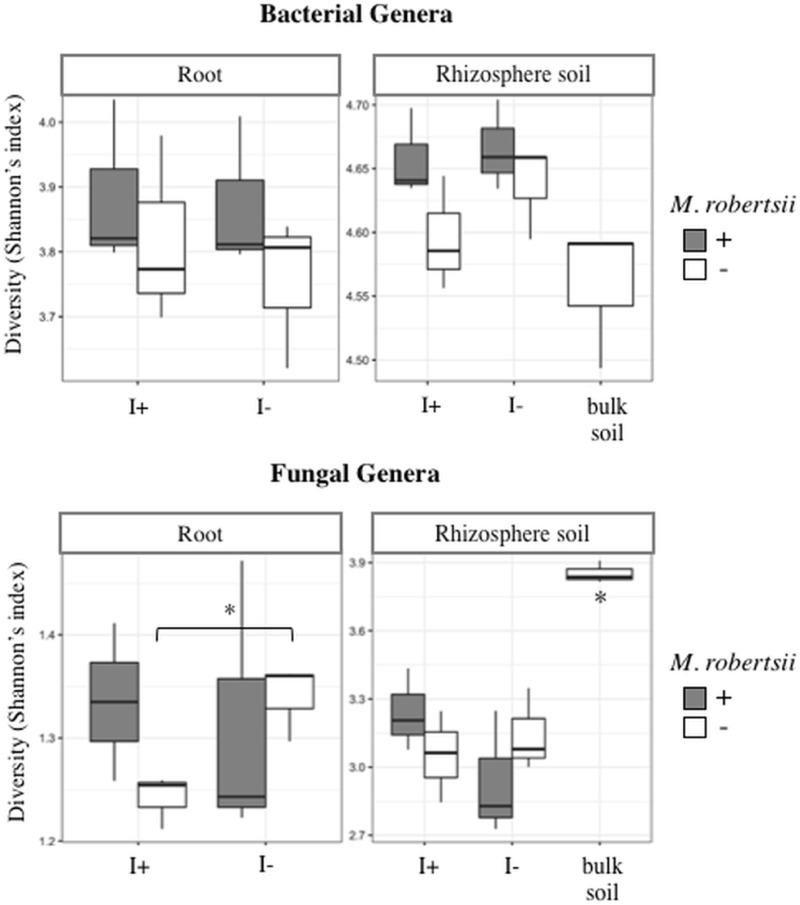
Shannon’s alpha diversity index between treatment groups for bacterial and fungal genera. I = insect larvae (*Galleria mellonella*). Asterisk denotes a significant difference (t-test, α = 0.05, n = 3).

To visualize microbiome similarities between the treatments, a principle coordinate analysis (PCoA) was performed on Bray-Curtis indexed OTUs (Fig 3). The PERMANOVA revealed significant separation between sample type (F = 18.83, *P* < 0.001) and treatment type (F = 1.82, *P* < 0.001). The treatments distinctly clustered based upon sample type (soil vs. root) and supported the dissimilar diversity that was visualized in Fig 2. Moreover, ANOVA analysis of the Shannon’s diversity (H’) revealed statistically higher bacterial diversity in the rhizospheric soil than in the root (F = 453.79, *P* < 0.001). However, the addition of *Metarhizium* did not result in significant changes to bacterial diversity, nor did any of the treatments (Fig 4, S1 Table). Similar trends were seen at the family and phylum level.

### *Metarhizium* effects on bacterial communities

**Fig 5:**
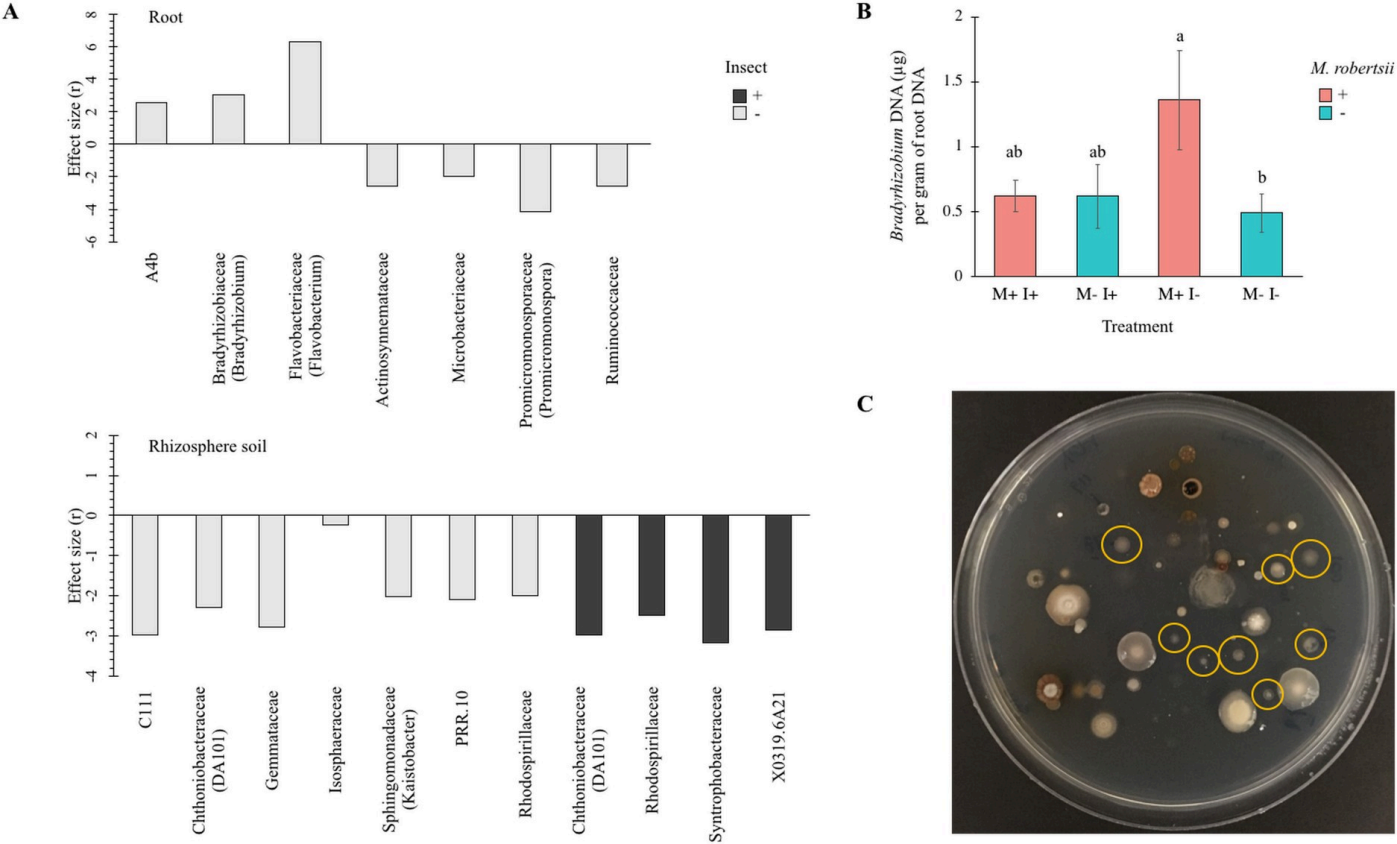
Bacteria significantly affected by *Metarhizium robertsii* amendment. The effect size from Welch’s t-tests using a corrected (fdr) P value (α = 0.05) is shown indicating relative abundance of bacterial Families (genus shown in brackets if known) significantly affected by *Metarhizium robertsii* amendment in (A) rhizosphere soil and root samples. Light bars = absence of larvae; dark bars = larvae present (n = 3). (B) The significant increase in the abundance of *Bradyrhizobium* in *Metarhizium* treated versus untreated root samples was confirmed with a SYBR® green PCR assay using *Bradyrhizobium*-specific primers. Error bars are standard deviation (n = 3). Conditions with different letters are significantly different (Scheffe’s post-hoc, α = 0.05). M: *M*. *robertsii* 2575-GFP. I: Insect, *Galleria mellonella*. (C) *Bradyrhizobium* (circled in yellow) cultured from potting soil on selective yeast-mannitol agar.

Welch’s t-tests were performed to examine if *Metarhizium* amendment significantly affected the abundance of specific bacteria (Fig 5). Fig 5 shows the significant (α = 0.05) increase or decrease of bacterial Families (identified genera shown in brackets) after *M*. *robertsii* addition, in rhizospheric soil and root tissue. The P values were corrected for false-discovery rate (fdr). Summarized statistical analyses are available in S5 Table.

Rhizospheric soil had a total of 11 (11/517 = 2.1%) bacterial taxa whose abundance was significantly reduced by *Metarhizium* amendment. There were seven OTUs significantly affected when *Galleria* larvae were absent (Fig 5A), belonging to a variety of phyla including Actinobacteria, Chloroflexi, Latescibacteria (previously WS3), Planctomycetes, Proteobacteria, and Verrucomicrobia. Only two of these OTUs were able to be identified to genus level and these were *Kaistobacter* and *DA101*. Within rhizospheric soil treated with a combination of *Galleria* larvae and fungal inoculum, there was a total of four bacterial families that were significantly affected (Fig 5A), Rhodospirillaceae, Syntrophobacteraceae, X0319.6A21, and Chthoniobacteraceae (genus DA101). In all cases, the relative abundance of these bacteria decreased significantly after *Metarhizium* amendment.

In root tissue there was a lower number of bacteria whose abundance was significantly affected by *Metarhizium* amendment (7/400 = 1.8%) (Fig 5A) and these changes only occurred in the absence of *Galleria* larvae. Actinosynnemataceae, Microbacteriaceae, Promicromonosporaceae (genus *Promicromonospora*), and Ruminococcaceae were reduced in root samples of beans grown in *Metarhizium*-amended soil. The relative abundance of Bradyrhizobiaceae, A4b, and Flavobacteriaceae (genus *Flavobacterium*) increased after fungal treatment. *Flavobacterium* was increased approximately 4-fold in *Metarhizium*-treated roots. An unclassified Bradyrhizobiaceae (genus *Bradyrhizobium* matched through BLASTn analysis) and unclassified A4b were not detected in root tissue of uninoculated bean plants but were present in root tissue of beans grown in *Metarhizium*-treated soil (Fig 5A). The increase in *Bradyrhizobium* was confirmed with qPCR using genus-specific primers [36] in a SYBR® green assay (Fig 5B). *Bradyrhizobium* was cultured on a selective medium [44] to confirm its detection was not from artificial DNA contamination [37] (Fig 5C).

### Identification and diversity of fungi in treatments

**Fig 6:**
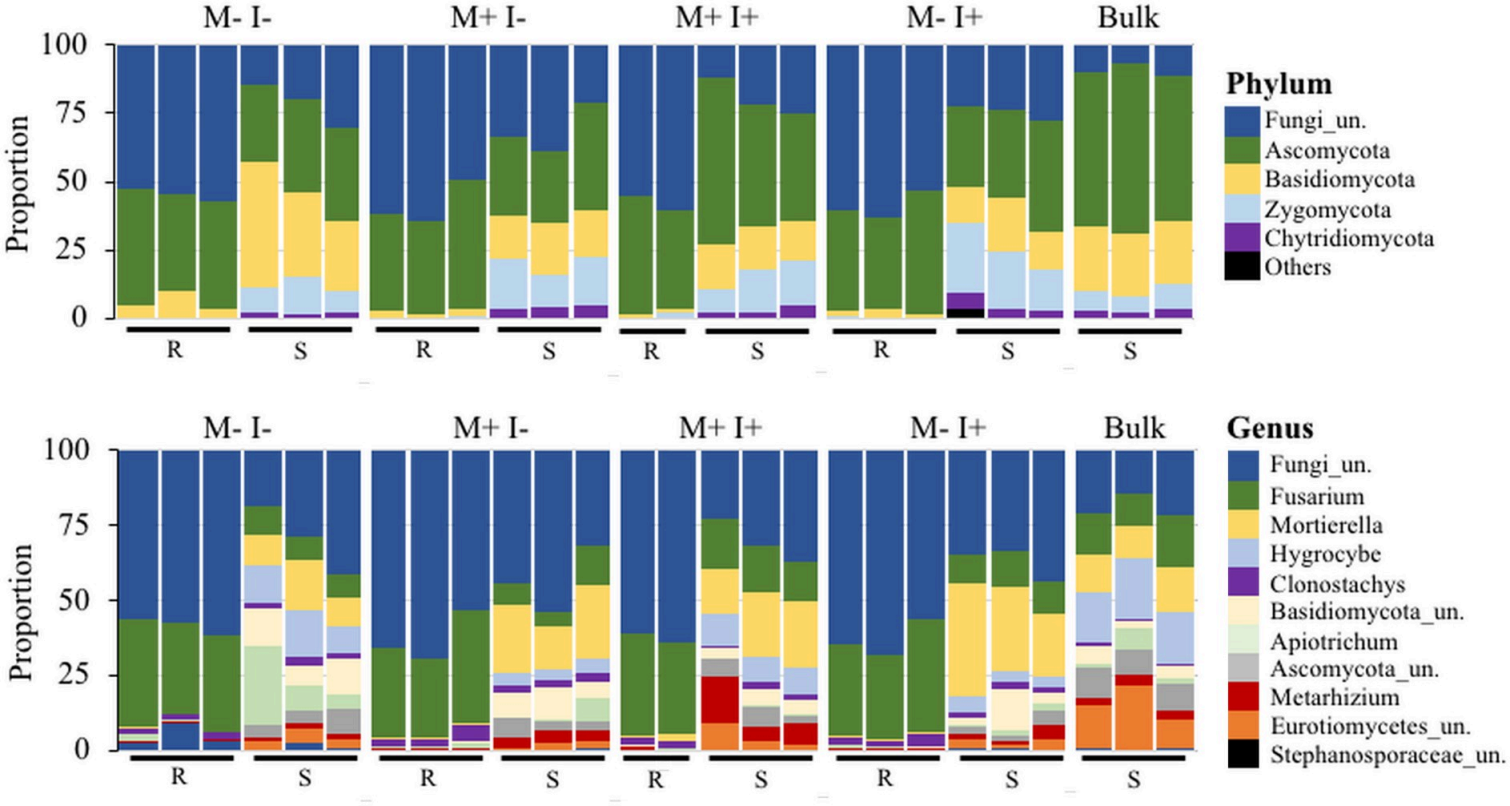
Proportional distribution of major (abundance greater than 1%) fungal phyla and genera present in *P*. *vulgaris* root (R) and rhizosphere soil (S). M: *M*. *robertsii* 2575-GFP. I: Insect, *Galleria mellonella*. Bulk soil represents samples of the original potting substrate (no bean plant). All conditions have three replicates which are pools of three samples each. “Others” signifies lower-abundance taxa.

A total of 446 fungal OTUs were identified through Illumina sequencing, with 42 and 47 enriched in the root and rhizosphere soil compartments, respectively (Fig 1C). The list of OTUs and the corresponding taxonomic classification can be viewed in S2 Table. The proportional abundance of the major fungal phyla and genera (abundance greater than 1%) is shown in Fig 6. In root samples, unclassified fungi accounted for approximately half of all OTUs at the phylum level with the remainder being mainly Ascomycota (Fig 6). In rhizospheric soil, the abundance of unclassified fungi was lower than in the root and was in approximately equal proportion to Ascomycota, Basidiomycota, and Zygomycota. Within the distribution of fungal genera, there remained a high proportion of unclassified fungi in root (~61%) and rhizospheric soil (~34%). In addition, root samples were predominated by *Fusarium* (>30%) and *Clonostachys*. Rhizospheric soil appeared more diverse in all treatments in comparison to root samples and proportionally different when compared to field-collected soil that was without a bean plant (i.e. original bulk soil) (Fig 6). *Fusarium*, *Mortierella*, *Hygrocybe*, unclassified Basidiomycota/Ascomycota/Eurotiomyctes/ Stephanosporaceae, *Apiotrichum*, *Clonostachys*, and *Metarhizium* were all in high abundance in rhizospheric soil of all treatments. The highest abundances of *Metarhizium* were unsurprisingly the soil samples from amended treatments, however, *Metarhizium* was detected in all samples as seen in Fig 1 and the fungal OTU relative abundance matrix (See S1 File).

Ordination analysis using the abundance profile of fungal genera shows the treatments distinctly clustered based upon sample type, similar to bacterial taxa, in the Bray-Curtis indexed PCoA analysis (Fig 3). The PERMANOVA revealed significant separation between sample type (F = 70.59, *P* < 0.001) and treatment type (F = 3.83, *P* < 0.001). Unlike bacteria, fungal taxa of bulk soil and rhizosphere soil, from all treatments, clustered separately from each other indicating that the bean plant altered the fungal community profiles; all treatments with a bean plant had lower diversity (Fig 4). Interestingly, this separation driven by the variance of axis 2 [9.6%] does not result in any further separation of the root fungal communities.

Similar to bacterial diversity, the diversity of fungi in soil (H’ = 2.7–3.9) was higher than that of the root (H’ = 1.2–1.5) (ANOVA, F = 657.71, *P* < 0.001). Shannon’s diversity revealed complex effects on global fungal diversity depending on treatment and sample type (Fig 4, S1 Table). In root samples, when an insect was present, there was no change in diversity with the amendment of *Metarhizium* (t-test, *P* = 0.4), however, without *Metarhizium*, the addition of larvae significantly decreased diversity (t-test, *P* = 0.02). In comparison, the addition of larvae did not reduce diversity in rhizospheric soil (Fig 4). Overall, *Metarhizium* amendment did not significantly alter fungal diversity.

### *Metarhizium* effects on fungal communities

**Fig 7:**
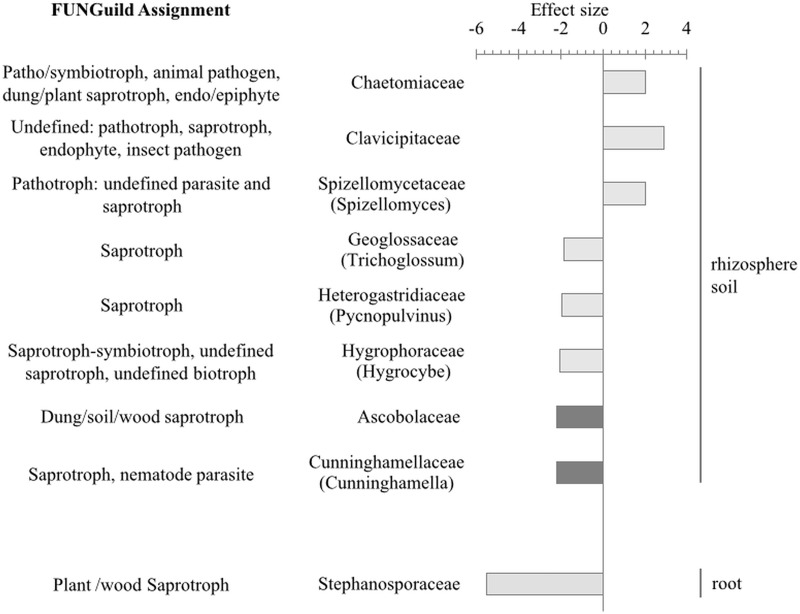
Fungi significantly affected by *Metarhizium robertsii* amendment. The effect size from Welch’s t-tests using a corrected (fdr) P value (α = 0.05) is shown indicating relative abundance of fungal Families (specific genus shown in brackets) significantly affected by *Metarhizium robertsii* amendment in rhizosphere soil and root samples. Light bars = absence of larvae; dark bars = larvae present (n = 3). Fungi were classified into lifestyle guilds using FUNGuild.

**Fig 8:**
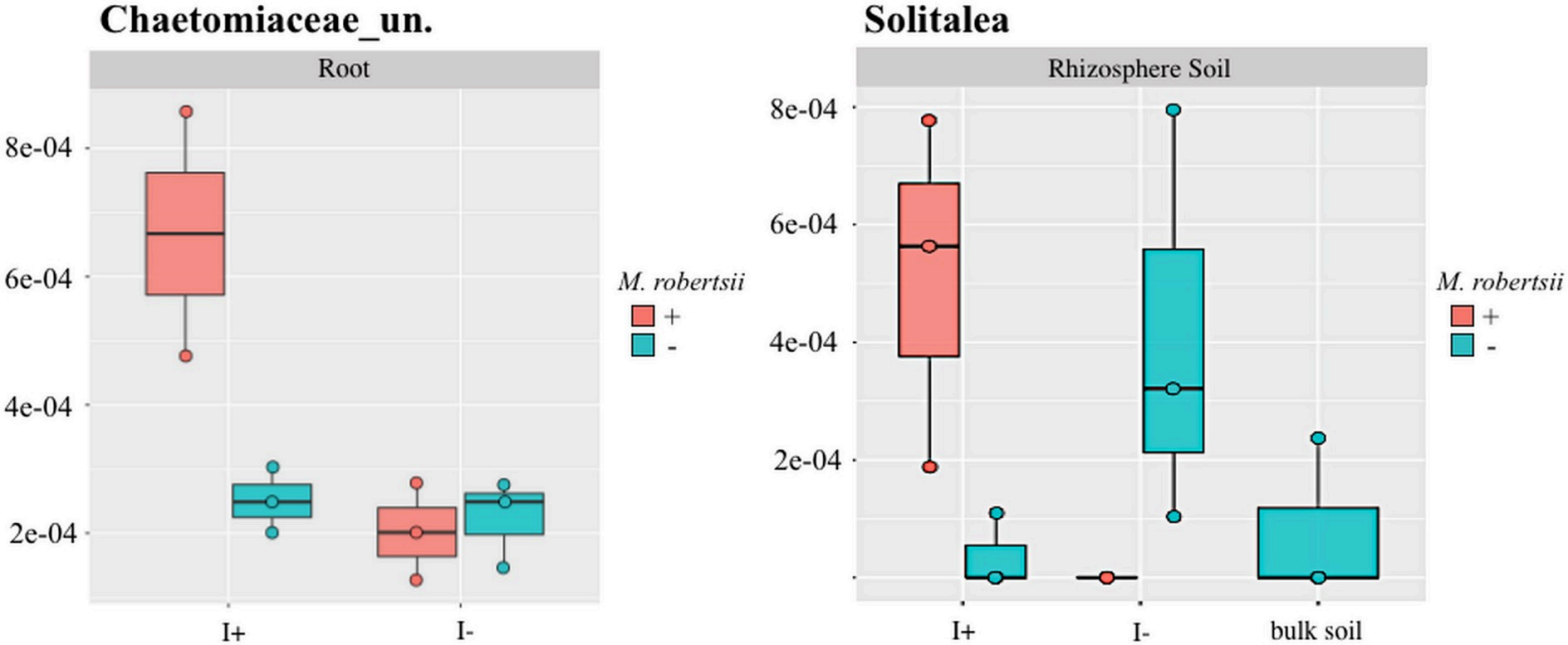
Interaction between the effect of adding *Metarhizium* and insect larvae as visualized by a box plot for *Solitalea* (bacteria) and Chaetomiaceae (fungi). The resultant effect on relative abundance from addition of fungus and insect together opposes the effect of either one applied individually. I = insect (*Galleria mellonella*) larvae. Bulk soil represents samples of the original potting substrate (no bean plant). Un. = unclassified.

Of the 384 identified fungal genera, pairwise comparisons showed that only 7 OTUs (7/259 = 2.7%) from rhizospheric soil and 1 OTU (1/361 = 0.3%) from root were significantly altered (fdr-adjusted, α = 0.05) upon *Metarhizium* amendment. Fig 8 shows the effect size, as calculated by distance-based Welch’s t-test, of fungal OTUs that could be classified to a minimum of family level, with a significant change in abundance after *M*. *robertsii* addition. FUNGuild assignment revealed that the majority of significantly affect taxa were saprotrophic (Fig 7). A summary of statistical analyses is available in S6 Table.

In the absence of *Galleria* larvae, a single fungal OTU from root tissue was significantly reduced by *Metarhizium* amendment, Stephanosporaceae (t-test, *P* = 0.01). In root samples from beans treated with a combination of *Galleria* larvae and *Metarhizium*, there were no OTUs that could be resolved to family level that were significantly affected.

*Metarhizium* amendment significantly increased the abundance of fungi in soil (in absence of larvae) from the families Clavicipitaceae, Chaetomiaceae, and Spizellomycetaceae (genus *Spizellomyces*) (Fig 7). The relative abundance of three unclassified species from the families Hygrophoraceae (*Hygrocybe*), Geoglossaceae (*Trichoglossum*), and Heterogastridiaceae (*Pycnopulvinus*) significantly decreased in rhizospheric soil from plants treated with *Metarhizium*; *Pycnopulvinus* was not detected in *Metarhizium-*amended samples.

Bean plants treated with a combination of *Metarhizium* amendment and *Galleria* larvae had a reduction in Cunninghamellaceae (*Cunninghamella*) (t-test, *P* = 0.04) and Ascobolaceae (t-test, *P* = 0.03) from rhizospheric soil (Fig 7). *Cunninghamella* was represented by a single OTU and BLAST analysis of the corresponding sequence revealed the species, *Cunninghamella elegans*.

### Metarhizium-galleria interaction

In addition to *Metarhizium* or the larvae amendments affecting microbe abundances, GLM analysis revealed that for some microorganisms there was an interaction when combining both treatments (S2 File, S3 and S4 Tables). In some cases, opposing effects were seen when either *Metarhizium* or *Galleria* were added independently compared to when they were combined in the treatment. An example of this is shown in Fig 8 with the bacteria *Solitalea* in root and an unclassified Chaetomiaceae (fungus) in rhizospheric soil. For *Solitalea*, when larvae were present the addition of *Metarhizium* resulted in an increase in abundance, whereas, when there were no larvae, the addition of *Metarhizium* resulted in a decrease in *Solitalea* (GLM, *P* = 0.01) (Fig 8). Similarly, in the root an unclassified Chaetomiaceae increased upon *Metarhizum* addition if larvae were also present, whereas there was no change in the abundance of Chaetomiaceae with the addition of either *Metarhizum* or larvae independently (GLM, *P* = 0.03) (Fig 8).

### Microbiome disease-suppressive activity

**Fig 9:**
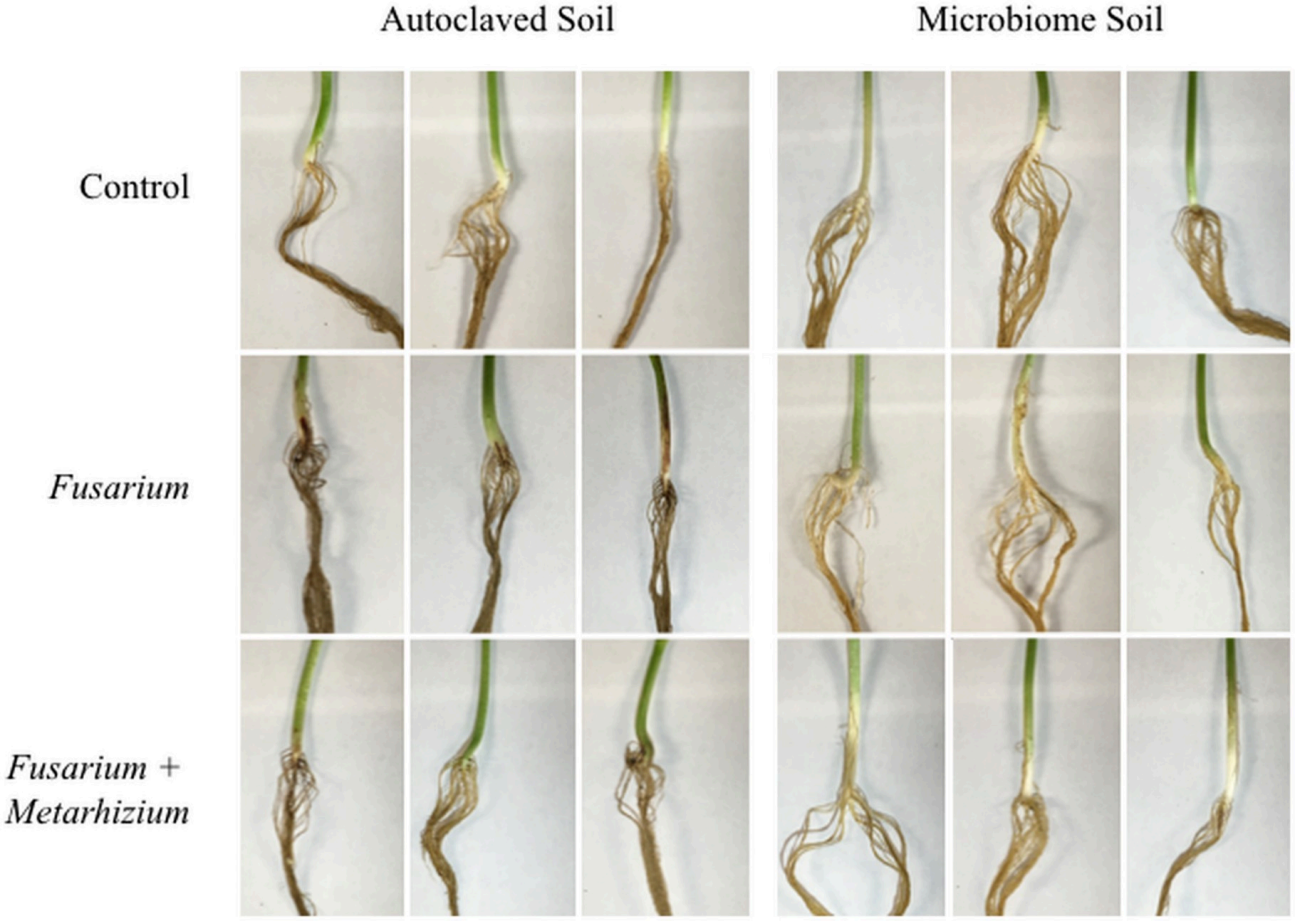
Evidence of disease-suppressive activity of the microbiome. Bean plants were treated with *Fusarium*, *Fusarium* and *Metarhizium*, or water, and grown for 14 days in microbiome soil or sterilized soil (autoclaved three times). Disease symptoms of root-rot in bean plants is seen as discolouration, lesions, and/or necrosis on the hypocotyl and/or roots. There were no indications of disease on the above-ground tissues. *Fusarium*: *Fusarium solani* f. sp. *phaseoli*; *Metarhizium*: *Metarhizium robertsii* 2575-GFP.

The capability of the microbiome to supress the action of a known fungal pathogen, *Fusarium solani* f. sp. *phaseoli*, to which *Metarhizium robertsii* has been shown to be antagonistic towards [23], was evaluated. Fig 9 shows the hypocotyl and upper root section for each treatment condition, as *F*. *solani* causes root-rot in *Phaseolus vulgaris* [41] and Table 2 shows the calculated disease scores. The hypocotyl of plants grown in autoclaved soil treated with *F*. *solani* showed the initial signs of disease. At 14 days, the hypocotyl and upper region of the taproot had reddish-brown, darkly discoloured lesions, indicating signs of necrosis (necrotic lesion index (NLI), 1.600 ± 0.414). These lesions were absent in *Fusarium*-treated plants that were grown in raw microbiome soil (NLI, 0.155 ± 0.042), indicating suppressive activity by the microbial community. Similarly, plants grown with *Metarhizium* in addition to *Fusarium* had noticeably less severe signs of disease (i.e. slight browning of upper tap root and tiny, sporadic lesions on hypocotyl) or completely lacked symptoms, in autoclaved soil (NLI, 0.450 ± 0.376). In microbiome soil, *Fusarium*-*Metarhizium* treated plants were free of any disease symptoms and were no different than control plants (NLI, 0.160 ± 0.047). There were no disease symptoms on above-ground tissues of any treatment. There was no difference in the dry weight of plants from any treatment (ANOVA, *P* = 0.94, n = 3) (S9 Table).

**Table 2:** Disease scoring of bean roots from disease-suppressive assay.

	Disease Indices^†^	
	Root rot index^‡^	Necrotic lesion index^§^
**Autoclaved soil**		
**Bean**	0.005 ± 0.004^a^	0.258 ± 0.163^a^
**Bean + *Fusarium***	0.225 ± 0.117^b^	1.600 ± 0.414^b^
**Bean + *Fusarium* + *Metarhizium***	0.040 ± 0.039^a^	0.450 ± 0.376^a^
**Microbiome soil**		
**Bean**	0.014 ± 0.011^a^	0.175 ± 0.065^a^
**Bean + *Fusarium***	0.020 ± 0.014^a^	0.155 ± 0.042^a^
**Bean + *Fusarium* + *Metarhizium***	0.020 ± 0.008^a^	0.160 ± 0.047^a^

^†^ Plants were treated with *Fusarium solani* f. sp. *phaseoli* with or without *Metarhizium robertsii* and grown in microbiome soil or autoclaved soil for 14 days. The average of each disease metric ± the standard deviation, is shown. Values followed by the same letter are not significantly different (Scheffe’s post hoc, α = 0.05; n = 3)

^‡^Dar *et al*. (1997) root rot disease index scored from 0 = no disease, to 1.00 = greater than 75% root area rotted.

^§^Filion *et al*. (2003) necrotic lesions scored from 0–5; 0 = no disease symptoms; 1 = slightly brown or <50% surface discoloration of the hypocotyl, firm upon pressure from thumb and forefinger, and slight root pruning; 2 = as 1 but >50% surface discoloration; 3 = discolored hypocotyl and roots collapsing under considerable pressure and extensive root pruning; 4 = darkly discolored hypocotyl and roots completely collapsed or collapsing easily under pressure and severe root pruning; 5 = dead or dying plant.

## Discussion

*M*. *robertsii* is the most abundant *Metarhizium* species isolated from farmland and open fields in Ontario [45]. Many studies have been conducted to determine its ability to persist in soil or to colonize plants [46–48] in order to determine ways to improve its survivability and thus, its utility as a biocontrol agent. Experimental trials under laboratory conditions often fail to translate to successful application under environmental settings. For example, in sterile laboratory conditions *M*. *brunneum* was capable of killing infected wireworms, however when implemented in the field, *M*. *brunneum* was suppressed by symbiotic bacteria [49] and rendered ineffective. Moreover, a study comparing endophytic colonization of *M*. *anisopliae* and *Beauveria bassiana* in bean plants showed unpredictable and highly variably levels in non-sterilized soil compared to sterile soil [50] indicating an influence from the microbial community. The success of EIPF agricultural amendments is constrained by a lack of understanding of the complex interactions within the existing microbiome of crops. This dynamic population can include organisms that will compete with or antagonize the applied biological control agent and thus limit its potential. This study presents an important step towards understanding the larger roles played by *Metarhizium* within its microbial community.

Diversity within a localized microbial community depends upon various mechanisms that restrict domination of the population by only a few competitive species [51]. Alterations that upset these mechanisms, whether biotic or abiotic factors, can lead to a decrease in diversity as a select few species become dominant; if these dominant organism(s) are pathogenic, plant disease develops [51]. However, a decrease in diversity is not always unfavourable. Plants shape the microbiome towards their specific requirements by means of their root exudate and thus rhizospheric soil is typically less diverse than the surrounding bulk soil [8,52,53]. The decrease in diversity of rhizosphere soil compared to bulk soil within this study likely reflects this recruitment of select organisms best suited to the needs of the growing plant.

*M*. *robertsii* amendment did not affect diversity and may be due to the presence of naturally occurring strains of *Metarhizium*. In contrast, fungal diversity of the root decreased when *Galleria mellonella* larvae were independently added to the soil (Fig 4). The inclusion of an insect to the system was to approximate naturally occurring tripartite *Metarhizium-*insect-plant interactions. Although *G*. *mellonella* is a parasite of beehives, thus not typically found in soil, it has been used consistently as a source of nitrogen for *Metarhizium* in nitrogen transfer experimentation [54]. Even in field-trials, *M*. *robertsii* was able to transfer insect-derived nitrogen to bean plants within 14 days post inoculation [21]. Insect presence in the system altered the overall composition of the microbiome and further amendment with *Metarhizium*, increasingly changed it structure. Specifically, there was an increase in the fungal species *Chaetomium*, *Trichoderma*, and an unclassified Chaetomiaceae which are ecologically important endophytic, plant growth promoting fungi. *Chaetomium* helps to support tolerance to copper toxicity in maize [55] and is antagonistic to plant pathogens such as apple scab [56]. The plant growth promoting effects of *Trichoderma* are numerous and include conferring tolerance to salinity stress, in wheat [57], suppression of root knot nematodes [58], induction of plant systemic resistance [59], and increased biomass and yield of soybean [60]. These tripartite interactions between plant-*Metarhizium-*insect require further experimentation to elucidate the total ecophysiological effects of these community changes within this system.

*Metarhizium* will persist in the rhizosphere of numerous plant species, often as a mutualistic endophyte [20,21,61]. Benefits of *Metarhizium* root colonization include tolerance to salt stress [18], increased biomass [19], and antagonism towards a fungal pathogen [23]. In the environment, disease suppression is a phenomenon in which the total microbial community prevents or suppresses the ability of pathogens to grow to sufficient number to infect their host [62]. Suppression arising from the action of a certain organism is “specific” suppression. For example, high abundance of *Pseudomonas* spp. in soil is suppressive to take-all disease of wheat by the fungal pathogen *Gaeumannomyces gramminis* var. *tritici* through the action of the antifungal compound 2,4-diacetylphloroglucinol (DAPG) [40]. Production of secondary metabolites can also act indirectly, such as lytic enzymes from Proteobacteria that specifically target a phytotoxin from *F*. *oxysporum* (fusaric acid) preventing its interaction with tomato plants and causing wilt [63]. Cell-free culture extracts from *Metarhizium* were shown to antagonize the growth of *F*. *solani* while the specific bioactive metabolite has yet to be identified [23]. Although non-pathogenic strains exist, *F*. *oxysporum* and *F*. *solani* are commonly plant root pathogens [14,64–66]. Both species were identified in the bean root by Illumina sequencing (S10 Table), yet all bean plants were healthy and free of disease symptoms (i.e. root discolouration/necrosis, leaf spots; data not shown) [41] which is indicative of disease suppression. However, even species-level identification is often insufficient to identify the lifestyle of a given organism, as evident by the variable FUNGuild classification of the fungi detected in this study. Towards this end, we challenged the microbiome with the known bean-specific pathogen, *Fusarium solani* f. sp. *phaseoli*. The phenomenon of disease-suppression by the microbial community was evident as *Fusarium* was unable to cause hypocotyl and upper root discolouration/necrosis when plants were grown in microbiome soil. Even at this early time point, disease symptoms were apparent in roots grown in sterilized soil; a condition greatly reduced or absent with application of *Metarhizium*. These results support the work done previously by Sasan and Bidochka (2013), who showed that *Metarhizium* antagonized *F*. *solani* f. sp. *phaseoli* in dual plate culture, in broth culture and *in planta*. The formation of root-rot disease symptoms observed by Sasan and Bidochka (2013) was more severe than we observed in our study (disease index 3.93 ± 0.07 and 1.60 ± 0.41, respectively) as their results were obtained after 4 weeks. Given that native *Metarhizium* spp. were present in all samples, it is likely that *Metarhizium* plays an important role with respect to disease suppression in this system.

In combination with the action of ‘specific’ suppression, is the collective activity of the soil microbiome in competing with or antagonising pathogens. This type of community activity is termed “general” suppression [39]. A large classification of bacteria noted for their contributions to disease suppression are better known as plant growth promoting bacteria (PGPB); notable genera include *Agrobacterium*, *Arthrobacter*, *Bacillus*, *Burkholderia*, *Caulobacter*, *Flavobacterium*, *Pseudomonas*, *Rhizobium*, *Serratia*, and *Streptomyces* [67,68]. The majority of members of these genera have been shown to be PGPB however, there still remains the existence of intraspecies variation (symbiotic/pathogenic) that is discernable only by identification of the specific strain [69]. The role of each taxa is discussed as potential means of PGP based upon the referenced information but whose true functionality remains to be proven. The abundance of *Flavobacterium*, *Bradyrhizobium* and unclassified bacteria in the family A4b (class Anaerolineae) were significantly increased (Welch’s t-test, α = 0.05) in root tissues after *Metarhizium robertsii* amendment (Fig 5). *Agrobacterium*, *Arthrobacter*, *Bacillus*, *Burkholderia*, *Caulobacter*, *Pseudomonas*, *Rhizobium*, *Serratia*, and *Streptomyces* were present in all root and soil samples yet remained unaffected by *Metarhizium* treatment (S2 File). The ecological role of members of the bacterial family A4b remains to be determined however sequencing techniques have detected them in ectomycorrhizal roots [70] and the Anaerolineae lineage of Chloroflexi from which they reside are known anaerobic digesters [71]. *Flavobacterium* species have been found in diverse aquatic and terrestrial habitats and are frequently one of the most abundant taxa to be detected in rhizospheric soils of numerous plant species [72,73]. *Flavobacterium* are believed to play an important role in plant growth and protection [73]. *Flavobacterium* species possess numerous extracellular macromolecular degrading enzymes that facilitate organic matter turnover [74] that can be crucial during the initial stages of plant growth. In addition, these bacteria can synthesize plant-growth stimulating hormones [75] as well as biologically active compounds against plant pathogens, such as *Phytophthora capsici* of pepper [76]. *Flavobacterium* was detected in high abundance in all plants which is in agreement with previous reports that *Flavobacterium* colonization is relatively high during early plant development (14 days) [73,77]. However, given that the abundance of *Flavobacterium* was significantly increased in roots after *Metarhizium* amendment (Fig 5), using *Metarhizium* to facilitate an increase in *Flavobacterium* root colonization could be a more favorable strategy over directly applying *Flavobacterium* strains as they are also fish and opportunistic human pathogens [73].

*M*. *robertsii* may contribute to general disease suppression and plant growth promotion in concert with PGPB members of the community. Of note, it was found that *Bradyrhizobium* was significantly increased in roots after *Metarhizium* amendment (Fig 5). *Bradyrhizobium* are classically viewed as important PGPB that are capable of nodulation and nitrogen fixation for leguminous plants, however, the dominant ecotype in natural populations do not form these typical symbioses [78,79] and indeed no nodules were observed in this study (data not shown). One hypothesis for the ecological importance of non-nodulating *Bradyrhizobium* species is the ability to increase plant host fitness by suppressing overproduction of nodules by prolific nodulating strains that would otherwise be energetically costly to the plant [79]. Co-inoculation of nodulating and non-nodulating strains of *Bradyrhizobium* isolated from *Acmispon strigosus* revealed that non-nodulating strains competitively colonized *A*. *strigosus* and depending on strain combination, could reduce the fitness of nodulating symbionts [79]. The plant growth promoting ability of *Bradyrhizobium* has also been shown for wild rice, *Oryza breviligulata* [80], and indicates that their ecological role is greater than their classical taxonomy indicates.

The roots of plant are known to be capable of housing numerous endophytes. *Metarhizium* amendment increased the abundance of an unclassified Helotiales (family Incertae sedis) and an unclassified Xylariales isolated from roots. Fungi of these orders are typically aquatic hyphomycetes which function to increase nutrient cycling in streams but are now recognized to possess a dual lifestyle as endophytes of terrestrial plants [81,82]. Occupation of plant tissues and utilization of resources by endophytes can competitively exclude pathogenic organisms to the benefit of the host. Antagonists can also have a direct negative effect on plant pathogens. Species of the fungal genus *Spizellomyces* (*Phlyctochytrium*) are parasites of nematodes [83], oospores of *Sclerospora sorghi* [83] and *Peronspora tabacina* [84], and the potato wart pathogen, *Synchytrium endobioticum* [85], in addition to their saprotrophic role [86]. Here, an increased abundance of *Spizellomyces* in soil after *Metarhizium* amendment was observed. Taken together these results suggest that in addition to the benefits conferred directly by *Metarhizium* such as promotion of disease suppression (*Fusarium* root rot) and exchanging insect-derived nitrogen for photosynthate [22,54], *Metarhizium* may act indirectly by promoting and maintaining an environment rich in numerous PGPB, facilitating up-take of nutrients and minerals, and integrating into the microbial community thus maintaining general disease suppressive activity of the soil.

*Metarhizium* is a broad-range insect pathogen used as a biological control agent. Its ability to beneficially colonize plant roots and persist in the rhizosphere permit its stability and longevity as a treatment, and further understanding of its role in larger microbiome communities will enhance its use as an agricultural treatment. The plant microbiome has been revealed as an important determinant of plant health and productivity, and next generation sequencing has overcome the limitation of culture-based analyses allowing for comprehensive analyses of plant associated microbial communities. Identifying the specific bacteria and fungi that are linked to beneficial plant properties, and how these inter and intra kingdom interactions relate to plant health, will fuel the development of synthetic communities and disease-suppressive soils for use in agriculture. By focusing this research on multifaceted microbial species, such as endophytic, insect pathogenic fungi, comprehensive treatments can be developed to ensure plant health in numerous ways.

## Supporting information

S1 File Bacterial and fungal OTU abundance matrices at phylum, family, and genus level. (XLSX)

S2 File Interactive data analysis website. [https://metarhiz-microbiome.shinyapps.io/shiny/].
(PDF)

S3 File R-scripts for data analysis and shiny app creation. (PDF)

S1 Table Experimental treatment setup for microbiome analysis. (PDF)

S2 Table OTUs enriched in root and rhizosphere soil. (PDF)

S3 Table Summary of generalized linear model (GLM) of bacterial taxa significantly affected by treatment. (PDF)

S4 Table Summary of generalized linear model (GLM) of fungal taxa significantly affected by treatment. (PDF)

S5 Table Bacterial taxa significantly affected by *Metarhizium robertsii* amendment determined by Welch’s t-test. (PDF)

S6 Table Fungal taxa significantly affected by *Metarhizium robertsii*. (PDF)

S7 Table Bacterial taxa significantly affected by *Galleria mellonella* treatment determined by Welch’s t-test. (PDF)

S8 Table Fungal taxa significantly affected by *Galleria mellonella* treatment determined by Welch’s t-test. (PDF)

S9 Table Dry weight measurements for bean plants from disease-suppressive soil assay. (PDF)

S10 Table Proportional distribution of *Fusarium* species OTUs. (PDF)
