# Supplementary material for: Plant microbiome analysis after Metarhizium amendment reveals increases in abundance of plant growth-promoting organisms and maintenance of disease-suppressive soil
Source: PLoS One. 2020 Apr 10;15(4):e0231150. doi: 10.1371/journal.pone.0231150 (PMC7147777; doi:10.1371/journal.pone.0231150)
Supplement: S2 File — [https://metarhiz-microbiome.shinyapps.io/shiny/]. (PDF) [file pone.0231150.s002.pdf]

S2 File. Interactive data analysis website.

Interactive data analysis website for common bean plant (*Phaseolus vulgaris*) root and soil microbiome alterations, after *Metarhizium robertsii* soil amendment. The generalized linear model (GLM) and Welch's t-tests can be adjusted for a desired P value, as well as for a particular factor of interest. A specific bacterial or fungal taxon can be selected to visualize the box plot of relative abundance and the corresponding significance testing.

https://metarhiz-microbiome.shinyapps.io/shiny3/

Available information for download:

- Raw reads (also available from Short Read Archive (NCBI), accession: PRJNA558088)
- OTU abundance tables for bacterial and fungal: phylum, family, and genus.
- R-script used for complete analysis
