# Supplementary material for: Plant microbiome analysis after Metarhizium amendment reveals increases in abundance of plant growth-promoting organisms and maintenance of disease-suppressive soil
Source: PLoS One. 2020 Apr 10;15(4):e0231150. doi: 10.1371/journal.pone.0231150 (PMC7147777; doi:10.1371/journal.pone.0231150)
Supplement: S3 File — (PDF) [file pone.0231150.s003.pdf]

```
#Microbiome analyses
# ========================= LOAD REQUIRED PACKAGES, DATA AND FUNCTIONS
####
### installing development version to handle two factors
#devtools::install_github("ggloor/ALDEx_bioc")
#  load necessary packages
library('shiny')
library('ggplot2')
library('reshape2')
library("data.table")
library("dplyr")
library("vegan")
library("gdata")
library("asbio") #for eveness
library('mvabund')
library("ALDEx2")
#
#
#setwd("/Users/alisonwaller/Documents/Professional/Brock/
Bidochka_Microbiome/shiny2/")
# start with with microbial insights proportions and multiply by that
Samples total reads
# to get back to reads
## proportions to reads
# Load experimental data (abundance tables)
# first row is the header, and first column is rownames (ie. doesn't
need corresponding column name)
f_genus<-read.table("b_Genus.csv",header=T,sep=",",row.names=1)
f_family<-read.table("b_Family.csv",header=T,sep=",",row.names=1)
f_phylum<-read.table("b_Phylum.csv",header=T,sep=",",row.names=1)
# read in bacterial data
bact_genus<-read.table("ss_Genus.csv",header=T,sep=",",row.names=1)
bact_family<-read.table("ss_Family.csv",header=T,sep=",",row.names=1)
bact_phylum<-read.table("ss_Phylum.csv",header=T,sep=",",row.names=1)
##
abundance_tables<-
list(fungi_genus=f_genus,fungi_family=f_family,fungi_phylum=f_phylum,

bact_genus=bact_genus,bact_family=bact_family,bact_phylum=bact_phylum)

## to get total reads per sample I need to read in the shared reads
files
fungi_shared_reads<-read.table("/Users/alisonwaller/Documents/
Professional/Brock/Bidochka_Microbiome/dataFromMicrobInsights/
fungi_counts_tax/
bidochka_reclust.trim.contigs.unique.good.precluster.pick.pick.agc.uni
que_list2.shared",
                               header=T,sep="\t")
#
fungi_shared_reads_dat<-fungi_shared_reads[!
```

```
grepl("Blank",fungi_shared_reads$Group),]
rownames(fungi_shared_reads_dat)<-fungi_shared_reads_dat$Group
rownames(fungi_shared_reads_dat)<-
sapply(strsplit(rownames(fungi_shared_reads_dat),"_"),"[[",1)
fungi_shared_reads_dat<-
fungi_shared_reads_dat[,grep("^Otu",colnames(fungi_shared_reads_dat))]
badSample<-"R-BFi-3"
fungi_shared_reads_dat<-fungi_shared_reads_dat[!
rownames(fungi_shared_reads_dat)%in%badSample,]
##
bact_shared_reads<-read.table("/Users/alisonwaller/Documents/
Professional/Brock/Bidochka_Microbiome/dataFromMicrobInsights/
bidchoka_ss.trim.contigs.unique.good.good.filter.unique.precluster.pic
k.pick.opti_mcc.unique_list.shared",
                              header=T,sep="\t")
#
bact_shared_reads_dat<-bact_shared_reads[!
grepl("Blank",bact_shared_reads$Group),]
rownames(bact_shared_reads_dat)<-bact_shared_reads_dat$Group
rownames(bact_shared_reads_dat)<-
sapply(strsplit(rownames(bact_shared_reads_dat),"_"),"[[",1)
bact_shared_reads_dat<-
bact_shared_reads_dat[,grep("^Otu",colnames(bact_shared_reads_dat))]
##
sample_metadata<-read.csv("sample_metadata.csv",row.names=1,header=T)
Sample_readTots_f<-rowSums(fungi_shared_reads_dat)
names(Sample_readTots_f)<-
sapply(strsplit(names(Sample_readTots_f),"_"),"[[",1)
Sample_readTots_b<-rowSums(bact_shared_reads_dat)
names(Sample_readTots_b)<-
sapply(strsplit(names(Sample_readTots_b),"_"),"[[",1)
sample_metadata$ReadTots_f<-
Sample_readTots_f[match(rownames(sample_metadata),names(Sample_readTot
s_f))]
sample_metadata$ReadTots_b<-
Sample_readTots_b[match(rownames(sample_metadata),names(Sample_readTot
s_b))]
##
prop2reads<-function(tab,ReadTots_f){
  metadata<-
sample_metadata[match(rownames(tab),rownames(sample_metadata)),]
  readTots<-metadata[,ReadTots]
  tab_reads<-round(tab*readTots)
}
# tR means calculated from total reads from that sample
f_genus_reads_tR<-prop2reads(f_genus,"ReadTots_f")
b_genus_reads_tR<-prop2reads(bact_genus,"ReadTots_b")
#
f_family_reads_tR<-prop2reads(f_family,"ReadTots_f")
b_family_reads_tR<-prop2reads(bact_family,"ReadTots_b")
```

```
#
f_phylum_reads_tR<-prop2reads(f_phylum,"ReadTots_f")
b_phylum_reads_tR<-prop2reads(bact_phylum,"ReadTots_b")
## subsetting functions ##
getBeanSub<-function(df){
  df<-df[grep("-B",rownames(df)),]
}
getSRSub<-function(df,SorR){
  df[grep(paste("^",SorR,sep=""),rownames(df)),]
}
#"Fungi"or"NF"
getFNFSub<-function(df,ForNF){
  samples<-rownames(subset(sample_metadata,Fungi==ForNF))
  df[intersect(samples,rownames(df)),]
}
#"Insect" or "NI
getINISub<-function(df,IorNI){
  samples<-rownames(subset(sample_metadata,Insect==IorNI))
  df[intersect(samples,rownames(df)),]
}
###### below here is old stuff starting with their proportion table
# Load experimental data (abundance tables)
# first row is the header, and first column is rownames (ie. doesn't
need corresponding column name)
##
abundance_tables_tR<-
list(fungi_genus=f_genus_reads_tR,fungi_family=f_family_reads_tR,fungi
_phylum=f_phylum_reads_tR,

bact_genus=b_genus_reads_tR,bact_family=b_family_reads_tR,bact_phylum=
b_phylum_reads_tR)
##
#df_name<-names(abundance_tables_tR)[1]
#df<-abundance_tables_tR[[df_name]]
writeAldexGlmTabs<-function(df_name){
  df<-abundance_tables_tR[[df_name]]
  df_B<-getBeanSub(df)
  #
  conditions<-sample_metadata[rownames(sample_metadata)
%in%rownames(df_B),c("Fungi","Insect")]
#  mm<- model.matrix(~ Fungi + Insect, conditions)
  mm.int <- model.matrix(~ Fungi * Insect, conditions)
#
#  x.FBS.mm <- aldex.clr(t(df.loc), mm, mc.samples=16, denom="all",
verbose=FALSE)
#  x.FBS.mm.glm<-aldex.glm(x.FBS.mm)
  x.FBS.mm.int <- aldex.clr(t(df_B), mm.int, mc.samples=256,
denom="all", verbose=FALSE)
  x.FBS.mm.int.glm<-aldex.glm(x.FBS.mm.int)
  cat(paste("writing glm table for: ", df_name))
```

```
write.table(x.FBS.mm.int.glm,paste(df_name,".mm.int.glm.txt",sep=""),s
ep="\t",row.names=T,col.names=NA)
}
sapply(names(abundance_tables_tR),function(x) writeAldexGlmTabs(x))
##
writeAldexGlmTabsByloc<-function(df_name){
  df<-abundance_tables_tR[[df_name]]
  df_B<-getBeanSub(df)
  locs<-c("S","R")
  for (i in 1:2){
    loc<-locs[i]
    df_Bloc<-getSRSub(df_B,loc)
  #
  conditions<-sample_metadata[rownames(sample_metadata)
%in%rownames(df_Bloc),c("Fungi","Insect")]
  #  mm<- model.matrix(~ Fungi + Insect, conditions)
  mm.int <- model.matrix(~ Fungi * Insect, conditions)
  #
  x.FBS.mm.int <- aldex.clr(t(df_Bloc), mm.int, mc.samples=256,
denom="all", verbose=FALSE)
  x.FBS.mm.int.glm<-aldex.glm(x.FBS.mm.int)
  cat(paste("writing glm table for: ", df_name,loc))

write.table(x.FBS.mm.int.glm,paste(df_name,".",loc,".mm.int.glm.txt",s
ep=""),sep="\t",row.names=T,col.names=NA)
  }
}
sapply(names(abundance_tables_tR),function(x)
writeAldexGlmTabsByloc(x))
##### now run tt-test analysis and export results
#df_names<-names(abundance_tables_tR)
#df_name<-df_names[1]
writeAldexTtTabs<-function(df_name){
  df<-abundance_tables_tR[[df_name]]
  dfB<-getBeanSub(df)
  #
  comp<-"FI"
  compOrder<-c("R-BFi-1","R-BFi-2","R-BFi-3","S-BFi-1","S-BFi-2","S-
BFi-3",
               "R-Bi-1","R-Bi-2","R-Bi-3","S-Bi-1","S-Bi-2","S-Bi-3")
  df.sub<-dfB[match(compOrder,rownames(dfB)),]
  # I have to do this as one sample is missing
  df.sub<-df.sub[complete.cases(df.sub),]
  conditions<-
sample_metadata[match(rownames(df.sub),rownames(sample_metadata)),c("F
ungi")]
  #df.sub<-dfB[grep("i",rownames(dfB)),]
  #conditions<-sample_metadata[rownames(sample_metadata)
%in%rownames(df.sub),c("Fungi")]
```

```
  df.subF.ald.tt<-aldex(t(df.sub), conditions, mc.samples=256, 
denom="all", test="t",effect=T,verbose=FALSE)

write.table(df.subF.ald.tt,paste(df_name,".",comp,".v2.tt.txt",sep="")
,sep="\t",row.names=T,col.names=NA)
  comp<-"FnI"
  compOrder<-c("R-BF-1","R-BF-2","R-BF-3","S-BF-1","S-BF-2","S-BF-3",
               "R-B1","R-B2","R-B3","S-B1","S-B2","S-B3")
  df.sub<-dfB[match(compOrder,rownames(dfB)),]
  # I have to do this as one sample is missing
  df.sub<-df.sub[complete.cases(df.sub),]
  conditions<-
sample_metadata[match(rownames(df.sub),rownames(sample_metadata)),c("F
ungi")]
  df.subF.ald.tt<-aldex(t(df.sub), conditions, mc.samples=256, 
denom="all", test="t",effect=T,verbose=FALSE)

write.table(df.subF.ald.tt,paste(df_name,".",comp,".v2.tt.txt",sep="")
,sep="\t",row.names=T,col.names=NA)
  comp<-"IF"
  compOrder<-c("R-BFi-1","R-BFi-2","R-BFi-3","S-BFi-1","S-BFi-2","S-
BFi-3",
                         "R-BF-1","R-BF-2","R-BF-3","S-BF-1","S-
BF-2","S-BF-3")
  df.sub<-dfB[match(compOrder,rownames(dfB)),]
  # I have to do this as one sample is missing
  df.sub<-df.sub[complete.cases(df.sub),]
  conditions<-
sample_metadata[match(rownames(df.sub),rownames(sample_metadata)),c("I
nsect")]
  df.subF.ald.tt<-aldex(t(df.sub), conditions, mc.samples=256, 
denom="all", test="t",effect=T,verbose=FALSE)

write.table(df.subF.ald.tt,paste(df_name,".",comp,".v2.tt.txt",sep="")
,sep="\t",row.names=T,col.names=NA)
  comp<-"InF"
  compOrder<-c("R-Bi-1","R-Bi-2","R-Bi-3","S-Bi-1","S-Bi-2","S-Bi-3",
                         "R-B1","R-B2","R-B3","S-B1","S-B2","S-B3")
  df.sub<-dfB[match(compOrder,rownames(dfB)),]
  # I have to do this as one sample is missing
  df.sub<-df.sub[complete.cases(df.sub),]
  conditions<-
sample_metadata[match(rownames(df.sub),rownames(sample_metadata)),c("I
nsect")]
  df.subF.ald.tt<-aldex(t(df.sub), conditions, mc.samples=256, 
denom="all", test="t",effect=T,verbose=FALSE)

write.table(df.subF.ald.tt,paste(df_name,".",comp,".v2.tt.txt",sep="")
,sep="\t",row.names=T,col.names=NA)
}
```

```
sapply(names(abundance_tables_tR),function(x) writeAldexTtTabs(x))
##
#df_names<-names(abundance_tables_tR)
#df_name<-df_names[1]
writeAldexTtTabsByloc<-function(df_name){
  df<-abundance_tables_tR[[df_name]]
  dfB<-getBeanSub(df)
  locs<-c("S","R")
  for (i in 1:2){
    loc<-locs[i]
    dfB_loc<-getSRSub(dfB,loc)
   ###
   comp<-"FI"
   compOrder<-
paste(loc,c("BFi-1","BFi-2","BFi-3","Bi-1","Bi-2","Bi-3"),sep="-")
   df.sub<-dfB_loc[match(compOrder,rownames(dfB_loc)),]
   # I have to do this as one sample is missing
   df.sub<-df.sub[complete.cases(df.sub),]
   conditions<-
sample_metadata[match(rownames(df.sub),rownames(sample_metadata)),c("F
ungi")]
   df.subF.ald.tt<-aldex(t(df.sub), conditions, mc.samples=256,
denom="all", test="t",effect=T,verbose=FALSE)
   #df.subF.ald.tt["Metarhizium",]

write.table(df.subF.ald.tt,paste(df_name,".",comp,".",loc,".v2.tt.txt"
,sep=""),sep="\t",row.names=T,col.names=NA)
   ###
   comp<-"FnI"
   compOrder<-
paste(loc,c("BF-1","BF-2","BF-3","B1","B2","B3"),sep="-")
   df.sub<-dfB_loc[match(compOrder,rownames(dfB_loc)),]
   # I have to do this as one sample is missing
   df.sub<-df.sub[complete.cases(df.sub),]
   conditions<-
sample_metadata[match(rownames(df.sub),rownames(sample_metadata)),c("F
ungi")]
   df.subF.ald.tt<-aldex(t(df.sub), conditions, mc.samples=256,
denom="all", test="t",effect=T,verbose=FALSE)

write.table(df.subF.ald.tt,paste(df_name,".",comp,".",loc,".v2.tt.txt"
,sep=""),sep="\t",row.names=T,col.names=NA)
   ###
   comp<-"IF"
   compOrder<-
paste(loc,c("BFi-1","BFi-2","BFi-3","BF-1","BF-2","BF-3"),sep="-")
   df.sub<-dfB_loc[match(compOrder,rownames(dfB_loc)),]
   # I have to do this as one sample is missing
   df.sub<-df.sub[complete.cases(df.sub),]
   conditions<-
```

```
sample_metadata[match(rownames(df.sub),rownames(sample_metadata)),c("I
nsect")]
   df.subF.ald.tt<-aldex(t(df.sub), conditions, mc.samples=256,
denom="all", test="t",effect=T,verbose=FALSE)

write.table(df.subF.ald.tt,paste(df_name,".",comp,".",loc,".v2.tt.txt"
,sep=""),sep="\t",row.names=T,col.names=NA)
   ###
   comp<-"InF"
   compOrder<-
paste(loc,c("Bi-1","Bi-2","Bi-3","B1","B2","B3"),sep="-")
   df.sub<-dfB_loc[match(compOrder,rownames(dfB_loc)),]
   # I have to do this as one sample is missing
   df.sub<-df.sub[complete.cases(df.sub),]
   conditions<-
sample_metadata[match(rownames(df.sub),rownames(sample_metadata)),c("I
nsect")]
   df.subF.ald.tt<-aldex(t(df.sub), conditions, mc.samples=256,
denom="all", test="t",effect=T,verbose=FALSE)

write.table(df.subF.ald.tt,paste(df_name,".",comp,".",loc,".v2.tt.txt"
,sep=""),sep="\t",row.names=T,col.names=NA)
  }
}
sapply(names(abundance_tables_tR),function(x)
writeAldexTtTabsByloc(x))


#Microbiome app creating using Shiny app
# ========================= LOAD REQUIRED PACKAGES, DATA AND FUNCTIONS
####
### installing development version to handle two factors
#devtools::install_github("ggloor/ALDEx_bioc")
#  load necessary packages
library('shiny')
library('ggplot2')
library('ggpubr')
library('gridExtra')
library('reshape2')
library('rsconnect')
library("vegan")
#devtools::install_github("ggloor/ALDEx_bioc")
#library("ALDEx2")  # don't need now as aldex tables are precomputed
#
#setwd("/Users/alisonwaller/Documents/Professional/Brock/
Bidochka_Microbiome/shiny3/")
# took too long to run aldex within app so Load pre-calculated results
from ttests and glm analysis
# first row is the header, and first column is rownames (ie. doesn't
need corresponding column name)
```

```
f_genus_ald<-
read.table("fungi_genus.mm.int.glm.txt",header=T,sep="\t",row.names=1)
f_family_ald<-
read.table("fungi_family.mm.int.glm.txt",header=T,sep="\t",row.names=1
)
f_phylum_ald<-
read.table("fungi_phylum.mm.int.glm.txt",header=T,sep="\t",row.names=1
)
# read in bacterial data
bact_genus_ald<-
read.table("bact_genus.mm.int.glm.txt",header=T,sep="\t",row.names=1)
bact_family_ald<-
read.table("bact_family.mm.int.glm.txt",header=T,sep="\t",row.names=1)
bact_phylum_ald<-
read.table("bact_phylum.mm.int.glm.txt",header=T,sep="\t",row.names=1)
##
aldex_tables<-
list(fungi_genus=f_genus_ald,fungi_family=f_family_ald,fungi_phylum=f_
phylum_ald,

bact_genus=bact_genus_ald,bact_family=bact_family_ald,bact_phylum=bact
_phylum_ald)
### for rhizospheric soil samples
fS_genus_ald<-
read.table("fungi_genus.S.mm.int.glm.txt",header=T,sep="\t",row.names=
1)
fS_family_ald<-
read.table("fungi_family.S.mm.int.glm.txt",header=T,sep="\t",row.names
=1)
fS_phylum_ald<-
read.table("fungi_phylum.S.mm.int.glm.txt",header=T,sep="\t",row.names
=1)
# read in bacterial data
bactS_genus_ald<-
read.table("bact_genus.S.mm.int.glm.txt",header=T,sep="\t",row.names=1
)
bactS_family_ald<-
read.table("bact_family.S.mm.int.glm.txt",header=T,sep="\t",row.names=
1)
bactS_phylum_ald<-
read.table("bact_phylum.S.mm.int.glm.txt",header=T,sep="\t",row.names=
1)
##
aldex_tables_S<-
list(fungi_genus=fS_genus_ald,fungi_family=fS_family_ald,fungi_phylum=
fS_phylum_ald,

bact_genus=bactS_genus_ald,bact_family=bactS_family_ald,bact_phylum=ba
ctS_phylum_ald)
### for root samples
```

```
fR_genus_ald<-
read.table("fungi_genus.R.mm.int.glm.txt",header=T,sep="\t",row.names=
1)
fR_family_ald<-
read.table("fungi_family.R.mm.int.glm.txt",header=T,sep="\t",row.names
=1)
fR_phylum_ald<-
read.table("fungi_phylum.R.mm.int.glm.txt",header=T,sep="\t",row.names
=1)
# read in bacterial data
bactR_genus_ald<-
read.table("bact_genus.R.mm.int.glm.txt",header=T,sep="\t",row.names=1
)
bactR_family_ald<-
read.table("bact_family.R.mm.int.glm.txt",header=T,sep="\t",row.names=
1)
bactR_phylum_ald<-
read.table("bact_phylum.R.mm.int.glm.txt",header=T,sep="\t",row.names=
1)
##
aldex_tables_R<-
list(fungi_genus=fR_genus_ald,fungi_family=fR_family_ald,fungi_phylum=
fR_phylum_ald,

bact_genus=bactR_genus_ald,bact_family=bactR_family_ald,bact_phylum=ba
ctR_phylum_ald)

## ############## read in t-test tables
## FI comparison
f_genus_tt_FI<-
read.table("fungi_genus.FI.v2.tt.txt",header=T,sep="\t",row.names=1)
f_family_tt_FI<-
read.table("fungi_family.FI.v2.tt.txt",header=T,sep="\t",row.names=1)
f_phylum_tt_FI<-
read.table("fungi_phylum.FI.v2.tt.txt",header=T,sep="\t",row.names=1)
# read in bacterial data
bact_genus_tt_FI<-
read.table("bact_genus.FI.v2.tt.txt",header=T,sep="\t",row.names=1)
bact_family_tt_FI<-
read.table("bact_family.FI.v2.tt.txt",header=T,sep="\t",row.names=1)
bact_phylum_tt_FI<-
read.table("bact_phylum.FI.v2.tt.txt",header=T,sep="\t",row.names=1)
## FnI
f_genus_tt_FnI<-
read.table("fungi_genus.FnI.v2.tt.txt",header=T,sep="\t",row.names=1)
f_family_tt_FnI<-
read.table("fungi_family.FnI.v2.tt.txt",header=T,sep="\t",row.names=1)
f_phylum_tt_FnI<-
read.table("fungi_phylum.FnI.v2.tt.txt",header=T,sep="\t",row.names=1)
# read in bacterial data
```

```
bact_genus_tt_FnI<-
read.table("bact_genus.FnI.v2.tt.txt",header=T,sep="\t",row.names=1)
bact_family_tt_FnI<-
read.table("bact_family.FnI.v2.tt.txt",header=T,sep="\t",row.names=1)
bact_phylum_tt_FnI<-
read.table("bact_phylum.FnI.v2.tt.txt",header=T,sep="\t",row.names=1)
## IF
f_genus_tt_IF<-
read.table("fungi_genus.IF.v2.tt.txt",header=T,sep="\t",row.names=1)
f_family_tt_IF<-
read.table("fungi_family.IF.v2.tt.txt",header=T,sep="\t",row.names=1)
f_phylum_tt_IF<-
read.table("fungi_phylum.IF.v2.tt.txt",header=T,sep="\t",row.names=1)
# read in bacterial data
bact_genus_tt_IF<-
read.table("bact_genus.IF.v2.tt.txt",header=T,sep="\t",row.names=1)
bact_family_tt_IF<-
read.table("bact_family.IF.v2.tt.txt",header=T,sep="\t",row.names=1)
bact_phylum_tt_IF<-
read.table("bact_phylum.IF.v2.tt.txt",header=T,sep="\t",row.names=1)
## InF
f_genus_tt_InF<-
read.table("fungi_genus.InF.v2.tt.txt",header=T,sep="\t",row.names=1)
f_family_tt_InF<-
read.table("fungi_family.InF.v2.tt.txt",header=T,sep="\t",row.names=1)
f_phylum_tt_InF<-
read.table("fungi_phylum.InF.v2.tt.txt",header=T,sep="\t",row.names=1)
# read in bacterial data
bact_genus_tt_InF<-
read.table("bact_genus.InF.v2.tt.txt",header=T,sep="\t",row.names=1)
bact_family_tt_InF<-
read.table("bact_family.InF.v2.tt.txt",header=T,sep="\t",row.names=1)
bact_phylum_tt_InF<-
read.table("bact_phylum.InF.v2.tt.txt",header=T,sep="\t",row.names=1)
#### creat list
f_genus_tt<-
list(FI=f_genus_tt_FI,FnI=f_genus_tt_FnI,IF=f_genus_tt_IF,InF=f_genus_
tt_InF)
f_family_tt<-
list(FI=f_family_tt_FI,FnI=f_family_tt_FnI,IF=f_family_tt_IF,InF=f_fam
ily_tt_InF)
f_phylum_tt<-
list(FI=f_phylum_tt_FI,FnI=f_phylum_tt_FnI,IF=f_phylum_tt_IF,InF=f_phy
lum_tt_InF)
##
bact_genus_tt<-
list(FI=bact_genus_tt_FI,FnI=bact_genus_tt_FnI,IF=bact_genus_tt_IF,InF
=bact_genus_tt_InF)
bact_family_tt<-
list(FI=bact_family_tt_FI,FnI=bact_family_tt_FnI,IF=bact_family_tt_IF,
```

```
InF=bact_family_tt_InF)
bact_phylum_tt<-
list(FI=bact_phylum_tt_FI,FnI=bact_phylum_tt_FnI,IF=bact_phylum_tt_IF,
InF=bact_phylum_tt_InF)
##
tt_tables<-
list(fungi_genus=f_genus_tt,fungi_family=f_family_tt,fungi_phylum=f_ph
ylum_tt,

bact_genus=bact_genus_tt,bact_family=bact_family_tt,bact_phylum=bact_p
hylum_tt)
### t-tables just soil
## FI comparison
f_genus_tt_FI_S<-
read.table("fungi_genus.FI.S.v2.tt.txt",header=T,sep="\t",row.names=1)
f_family_tt_FI_S<-
read.table("fungi_family.FI.S.v2.tt.txt",header=T,sep="\t",row.names=1
)
f_phylum_tt_FI_S<-
read.table("fungi_phylum.FI.S.v2.tt.txt",header=T,sep="\t",row.names=1
)
# read in bacterial data
bact_genus_tt_FI_S<-
read.table("bact_genus.FI.S.v2.tt.txt",header=T,sep="\t",row.names=1)
bact_family_tt_FI_S<-
read.table("bact_family.FI.S.v2.tt.txt",header=T,sep="\t",row.names=1)
bact_phylum_tt_FI_S<-
read.table("bact_phylum.FI.S.v2.tt.txt",header=T,sep="\t",row.names=1)
## FnI
f_genus_tt_FnI_S<-
read.table("fungi_genus.FnI.S.v2.tt.txt",header=T,sep="\t",row.names=1
)
f_family_tt_FnI_S<-
read.table("fungi_family.FnI.S.v2.tt.txt",header=T,sep="\t",row.names=
1)
f_phylum_tt_FnI_S<-
read.table("fungi_phylum.FnI.S.v2.tt.txt",header=T,sep="\t",row.names=
1)
# read in bacterial data
bact_genus_tt_FnI_S<-
read.table("bact_genus.FnI.S.v2.tt.txt",header=T,sep="\t",row.names=1)
bact_family_tt_FnI_S<-
read.table("bact_family.FnI.S.v2.tt.txt",header=T,sep="\t",row.names=1
)
bact_phylum_tt_FnI_S<-
read.table("bact_phylum.FnI.S.v2.tt.txt",header=T,sep="\t",row.names=1
)
## IF
f_genus_tt_IF_S<-
read.table("fungi_genus.IF.S.v2.tt.txt",header=T,sep="\t",row.names=1)
```

```
f_family_tt_IF_S<-
read.table("fungi_family.IF.S.v2.tt.txt",header=T,sep="\t",row.names=1
)
f_phylum_tt_IF_S<-
read.table("fungi_phylum.IF.S.v2.tt.txt",header=T,sep="\t",row.names=1
)
# read in bacterial data
bact_genus_tt_IF_S<-
read.table("bact_genus.IF.S.v2.tt.txt",header=T,sep="\t",row.names=1)
bact_family_tt_IF_S<-
read.table("bact_family.IF.S.v2.tt.txt",header=T,sep="\t",row.names=1)
bact_phylum_tt_IF_S<-
read.table("bact_phylum.IF.S.v2.tt.txt",header=T,sep="\t",row.names=1)
## InF
f_genus_tt_InF_S<-
read.table("fungi_genus.InF.S.v2.tt.txt",header=T,sep="\t",row.names=1
)
f_family_tt_InF_S<-
read.table("fungi_family.InF.S.v2.tt.txt",header=T,sep="\t",row.names=
1)
f_phylum_tt_InF_S<-
read.table("fungi_phylum.InF.S.v2.tt.txt",header=T,sep="\t",row.names=
1)
# read in bacterial data
bact_genus_tt_InF_S<-
read.table("bact_genus.InF.S.v2.tt.txt",header=T,sep="\t",row.names=1)
bact_family_tt_InF_S<-
read.table("bact_family.InF.S.v2.tt.txt",header=T,sep="\t",row.names=1
)
bact_phylum_tt_InF_S<-
read.table("bact_phylum.InF.S.v2.tt.txt",header=T,sep="\t",row.names=1
)
#### create lists
f_genus_tt_S<-
list(FI=f_genus_tt_FI_S,FnI=f_genus_tt_FnI_S,IF=f_genus_tt_IF_S,InF=f_
genus_tt_InF_S)
f_family_tt_S<-
list(FI=f_family_tt_FI_S,FnI=f_family_tt_FnI_S,IF=f_family_tt_IF_S,InF
=f_family_tt_InF_S)
f_phylum_tt_S<-
list(FI=f_phylum_tt_FI_S,FnI=f_phylum_tt_FnI_S,IF=f_phylum_tt_IF_S,InF
=f_phylum_tt_InF_S)
##
bact_genus_tt_S<-
list(FI=bact_genus_tt_FI_S,FnI=bact_genus_tt_FnI_S,IF=bact_genus_tt_IF
_S,InF=bact_genus_tt_InF_S)
bact_family_tt_S<-
list(FI=bact_family_tt_FI_S,FnI=bact_family_tt_FnI_S,IF=bact_family_tt
_IF_S,InF=bact_family_tt_InF_S)
bact_phylum_tt_S<-
```

```
list(FI=bact_phylum_tt_FI_S,FnI=bact_phylum_tt_FnI_S,IF=bact_phylum_tt
_IF_S,InF=bact_phylum_tt_InF_S)
##
tt_tables_S<-
list(fungi_genus=f_genus_tt_S,fungi_family=f_family_tt_S,fungi_phylum=
f_phylum_tt_S,

bact_genus=bact_genus_tt_S,bact_family=bact_family_tt_S,bact_phylum=ba
ct_phylum_tt_S)
## t-test tables just Root
## FI comparison
f_genus_tt_FI_R<-
read.table("fungi_genus.FI.R.v2.tt.txt",header=T,sep="\t",row.names=1)
f_family_tt_FI_R<-
read.table("fungi_family.FI.R.v2.tt.txt",header=T,sep="\t",row.names=1
)
f_phylum_tt_FI_R<-
read.table("fungi_phylum.FI.R.v2.tt.txt",header=T,sep="\t",row.names=1
)
# read in bacterial data
bact_genus_tt_FI_R<-
read.table("bact_genus.FI.R.v2.tt.txt",header=T,sep="\t",row.names=1)
bact_family_tt_FI_R<-
read.table("bact_family.FI.R.v2.tt.txt",header=T,sep="\t",row.names=1)
bact_phylum_tt_FI_R<-
read.table("bact_phylum.FI.R.v2.tt.txt",header=T,sep="\t",row.names=1)
## FnI
f_genus_tt_FnI_R<-
read.table("fungi_genus.FnI.R.v2.tt.txt",header=T,sep="\t",row.names=1
)
f_family_tt_FnI_R<-
read.table("fungi_family.FnI.R.v2.tt.txt",header=T,sep="\t",row.names=
1)
f_phylum_tt_FnI_R<-
read.table("fungi_phylum.FnI.R.v2.tt.txt",header=T,sep="\t",row.names=
1)
# read in bacterial data
bact_genus_tt_FnI_R<-
read.table("bact_genus.FnI.R.v2.tt.txt",header=T,sep="\t",row.names=1)
bact_family_tt_FnI_R<-
read.table("bact_family.FnI.R.v2.tt.txt",header=T,sep="\t",row.names=1
)
bact_phylum_tt_FnI_R<-
read.table("bact_phylum.FnI.R.v2.tt.txt",header=T,sep="\t",row.names=1
)
## IF
f_genus_tt_IF_R<-
read.table("fungi_genus.IF.R.v2.tt.txt",header=T,sep="\t",row.names=1)
f_family_tt_IF_R<-
read.table("fungi_family.IF.R.v2.tt.txt",header=T,sep="\t",row.names=1
```

```
)
f_phylum_tt_IF_R<-
read.table("fungi_phylum.IF.R.v2.tt.txt",header=T,sep="\t",row.names=1
)
# read in bacterial data
bact_genus_tt_IF_R<-
read.table("bact_genus.IF.R.v2.tt.txt",header=T,sep="\t",row.names=1)
bact_family_tt_IF_R<-
read.table("bact_family.IF.R.v2.tt.txt",header=T,sep="\t",row.names=1)
bact_phylum_tt_IF_R<-
read.table("bact_phylum.IF.R.v2.tt.txt",header=T,sep="\t",row.names=1)
## InF
f_genus_tt_InF_R<-
read.table("fungi_genus.InF.R.v2.tt.txt",header=T,sep="\t",row.names=1
)
f_family_tt_InF_R<-
read.table("fungi_family.InF.R.v2.tt.txt",header=T,sep="\t",row.names=
1)
f_phylum_tt_InF_R<-
read.table("fungi_phylum.InF.R.v2.tt.txt",header=T,sep="\t",row.names=
1)
# read in bacterial data
bact_genus_tt_InF_R<-
read.table("bact_genus.InF.R.v2.tt.txt",header=T,sep="\t",row.names=1)
bact_family_tt_InF_R<-
read.table("bact_family.InF.R.v2.tt.txt",header=T,sep="\t",row.names=1
)
bact_phylum_tt_InF_R<-
read.table("bact_phylum.InF.R.v2.tt.txt",header=T,sep="\t",row.names=1
)
#### creat list
f_genus_tt_R<-
list(FI=f_genus_tt_FI_R,FnI=f_genus_tt_FnI_R,IF=f_genus_tt_IF_R,InF=f_
genus_tt_InF_R)
f_family_tt_R<-
list(FI=f_family_tt_FI_R,FnI=f_family_tt_FnI_R,IF=f_family_tt_IF_R,InF
=f_family_tt_InF_R)
f_phylum_tt_R<-
list(FI=f_phylum_tt_FI_R,FnI=f_phylum_tt_FnI_R,IF=f_phylum_tt_IF_R,InF
=f_phylum_tt_InF_R)
##
bact_genus_tt_R<-
list(FI=bact_genus_tt_FI_R,FnI=bact_genus_tt_FnI_R,IF=bact_genus_tt_IF
_R,InF=bact_genus_tt_InF_R)
bact_family_tt_R<-
list(FI=bact_family_tt_FI_R,FnI=bact_family_tt_FnI_R,IF=bact_family_tt
_IF_R,InF=bact_family_tt_InF_R)
bact_phylum_tt_R<-
list(FI=bact_phylum_tt_FI_R,FnI=bact_phylum_tt_FnI_R,IF=bact_phylum_tt
_IF_R,InF=bact_phylum_tt_InF_R)
```

```
##
tt_tables_R<-
list(fungi_genus=f_genus_tt_R,fungi_family=f_family_tt_R,fungi_phylum=
f_phylum_tt_R,

bact_genus=bact_genus_tt_R,bact_family=bact_family_tt_R,bact_phylum=ba
ct_phylum_tt_R)

#### read in proportions for plot
f_genus<-read.table("b_Genus.csv",header=T,sep=",",row.names=1)
f_family<-read.table("b_Family.csv",header=T,sep=",",row.names=1)
f_phylum<-read.table("b_Phylum.csv",header=T,sep=",",row.names=1)
# read in bacterial data
bact_genus<-read.table("ss_Genus.csv",header=T,sep=",",row.names=1)
bact_family<-read.table("ss_Family.csv",header=T,sep=",",row.names=1)
bact_phylum<-read.table("ss_Phylum.csv",header=T,sep=",",row.names=1)
# sample metadata
sample_metadata<-read.csv("sample_metadata.csv",row.names=1,header=T)
##
abundance_tables<-
list(fungi_genus=f_genus,fungi_family=f_family,fungi_phylum=f_phylum,

bact_genus=bact_genus,bact_family=bact_family,bact_phylum=bact_phylum)
#"Fungi"or"NF"
getFNFSub<-function(df,ForNF){
  samples<-rownames(subset(sample_metadata,Fungi==ForNF))
  df[intersect(samples,rownames(df)),]
}
#"Insect" or "NI
getINISub<-function(df,IorNI){
  samples<-rownames(subset(sample_metadata,Insect==IorNI))
  df[intersect(samples,rownames(df)),]
}
##
FactorsOfInt<-c("M.robertii","Insect","M.robertii*Insect")
CompOfInt<-c("M+I+ vs M-I+","M+I- vs M-I-","I+M+ vs I-M+","I+M- vs I-
M-")
Indices<-c("Diversity_sh","Diversity_si","Evenness","Richness_chao1")
# ui setting up user interface
#####
ui <- fluidPage(
  # Make a title to display in the app
  titlePanel(" Exploring the Effect of Metarhizium on the Soil and
Root Microbiome "),
  # Make the Sidebar layout
  sidebarLayout(
    # Put in the sidebar all the input functions
    sidebarPanel(
      tabsetPanel(id="tabs",
                  tabPanel("otu",br(),
```

```
                           p("On this tab you can choose to look at
results from glm analysis and pair-wise tests for otus' of choice\n
                             first choose your dataset (either
bacterial or fungal, and the phylogenetic resolution),then the
specific otu"),
                           selectInput('datasetO', 'dataset',
names(abundance_tables),selected=names(abundance_tables)[1]),
                           uiOutput("otu"), br(),
                           # Add comment
                           p("For details on OTU identification please
refer to the original publications")),
                  tabPanel("diversity",br(),
                           p("On this tab you can visulize differnces
in diversity measures \n
                             first choose your dataset (either
bacterial or fungal, and the phylogenetic resolution),then the
specific otu"),
                           selectInput('datasetDiv', 'dataset',
names(abundance_tables),selected=names(abundance_tables)[1]),
                           selectInput('index','index of
interest',Indices,selected="Diversity_sh")),
                           # Add comment
                  tabPanel("glm", br(),
                           p("On this tab you can view results og
generalized linear models, choose a p-value cutoff and factors of
interest"),br(),
                           sliderInput('pval','p-value for
significance',

value=0.1,min=0,max=0.2,step=0.00001),
                           selectInput('datasetG', 'dataset',
names(abundance_tables)),
                           selectInput('FactorsOfInt','factor of
interest',FactorsOfInt,selected="M.robertii")),
                  tabPanel("ttests", br(),
                           p("On this tab you can view results of t-
test, choose a p-value cutoff and comparisons of interest"),br(),
                           sliderInput('pvalT','p-value for
significance',

value=0.1,min=0,max=0.5,step=0.00001),
                           selectInput('datasetT', 'dataset',
names(abundance_tables)),
                           selectInput('CompOfInt','comparison of
interest',CompOfInt,selected="M+I+ vs M-I+")),
                  tabPanel("downloads",selectInput('datasetD', 'raw
datasets', names(abundance_tables),selected=names(abundance_tables)
[1]),
                           downloadButton("downloadData",
"Download_raw-data"))
```

```
                  #
                  #tabPanel("downloads",selectInput('datasetD', 'raw
datasets', names(abundance_tables),selected=names(abundance_tables)
[1]),
                   #        downloadButton("downloadData",
"Download_raw-data"),
                    #       br(),
                      #     h4("Download scripts"),
                       #    downloadButton("downloadShiny",
"Download_Shiny_Script"),
                        #   br(),
                         #  downloadButton("downloadPreanal",
"Download_Pre-analysis"))
      )
    ),
    # Put in the main panel of the layout the output functions
    mainPanel(
      conditionalPanel(condition="input.tabs == 'otu'",
                       plotOutput('plot'),
                       h2("t-tests results for selected OTU"),
                       h3("by sample location"),
                       fluidRow(

column(strong("Root"),width=6,tableOutput("tabletR")),
                         column(strong("Rhizospheric
Soil"),width=6,tableOutput("tabletS"))
                       ),
                       h3("regardless of sample location"),
                       tableOutput("tablet"),
                       h2("glm results for selected OTU"),
                       h3("by sample location"),
                       fluidRow(

column(strong("Root"),width=6,tableOutput("tableR")),
                         column(strong("Rhizospheric
Soil"),width=6,tableOutput("tableS"))
                       ),
                       h3("regardless of sample location"),
                       tableOutput("table1")
      ),
      conditionalPanel(condition="input.tabs == 'diversity'",
                       h2("plots for different diversity indices"),
                       h3("Rhizospheric Soil"),
                       plotOutput("divplotS"),
                       h3("Root"),
                       plotOutput("divplotR"),
                       #fluidRow(
                      #
column(strong("Root"),width=6,plotOutput("divplotR")),
```

```
                       # column(strong("Rhizospheric
Soil"),width=6,plotOutput("divplotS"))
                      #  ),
                      br()
      ),
      conditionalPanel(condition="input.tabs == 'glm'",
                       #plotOutput('plot2')
                       br(),
                       h2("results for glm analysis for factor of
interest at selected significance"),
                       h3("by Sample Location"),
                       strong("Root"),
                       tableOutput("glm_tabR"),
                       strong("Rhizospheric Soil"),
                       tableOutput("glm_tabS"),
                       h3("all treatments"),
                       br(),
                       tableOutput("glm_tab2")
      ),
      conditionalPanel(condition="input.tabs == 'ttests'",
                       #plotOutput('plot2')
                       br(),
                       h2("results for t-tests by selected
significance"),
                       h3("by Sample Type"),
                       strong("Root"),
                       tableOutput("tt_tabR"),
                       strong("Rhizospheric Soil"),
                       tableOutput("tt_tabS"),
                       h3("all treatments"),
                       tableOutput("tt_tab2")
      )
    )
  )
)
####
# done setting up user interface
# ======================== SERVER ####
server <- function(input, output){
  # Return the requested dataset ----
  datasetNameO<-reactive({input$datasetO})
  datasetNameDiv<-reactive({input$datasetDiv})
 # datasetInput <- reactive({
#    abundance_tables[[input$dataset]]
#  })
  pvalInputG<-reactive({
    input$pval
  })
  FactorsInput<-reactive({
    input$FactorsOfInt
```

```
  })
  datasetNameG<-reactive({input$datasetG})
  #remove if no pw
  comparisonInput<-reactive({
    input$CompOfInt
  })
  pvalInputT<-reactive({
    input$pvalT
  })
  datasetNameT<-reactive({input$datasetT})
  # dataset chosen in download tab
  datasetNameD<-reactive({input$datasetD})
  # output otus to choose basaed on dataset selection
  output$otu <- renderUI({
    selectInput(inputId = "otu", label = "otu",
                choices =
colnames(abundance_tables[[datasetNameO()]]),selected="Metarhizium")
  })
  otuInput<-reactive({
    input$otu
  })
  indexInput<-reactive({
    input$index
  })
  ##
  output$plot <- renderPlot({
     req(is.null(input$otu)==F)
    df<-abundance_tables[[datasetNameO()]]
    otu<-otuInput()
    ## melt and add sample metadata
    df_annot<-merge(df,sample_metadata,by="row.names",all.x=T)
    rownames(df_annot)<-df_annot[,1]
    df_annot<-df_annot[,-1]
    #
   # df_annot<-subset(df_annot,df_annot$Bean =="Bean")
    #
    dfM<-melt(df_annot,id.vars =
c("Location","Bean","Fungi","Insect"),value.name="abund")
    # renaming Fungi level to metarhizium
    levels(dfM$Fungi)<-c("M+","M-")
    levels(dfM$Location)<-c("Root","Rhizospheric Soil")
    # subset based on otu of interest
    dfM.sub<-subset(dfM,dfM$variable==otu)
    dfM.sub$Insect[which(dfM.sub$Bean =="No bean")]<-"bulk_soil"
    #
    ggplot(dfM.sub,aes(x=Insect,y=abund,fill=Fungi))
+geom_boxplot(alpha=0.8)+geom_point(aes(fill=Fungi),size = 3, shape =
21,position = position_jitterdodge(jitter.width = 0.02,jitter.height =
0))+
      facet_wrap(~Location,scales="free" )+
```

```
      guides(fill=guide_legend("M. robertsii")) +
      ggtitle(otu)+
      scale_x_discrete(labels= c("I+","I-","bulk soil"))+
      ylab("proprotional abundance")+
      theme(plot.title = element_text(size = 18, face = "bold"))+
      theme(axis.text=element_text(size=14),
            axis.title=element_text(size=14)) +
      theme(legend.text=element_text(size=14),
            legend.title=element_text(size=14)) +
      theme(strip.text.x = element_text(size = 14))
  })
  ## diversity plots
  output$divplotR<-renderPlot({
    req(is.null(input$otu)==F)
    df<-abundance_tables_tR[[datasetNameDiv()]]
    index<-indexInput()
      ## Root first
      df_loc<-getSRSub(df,"R")
      df_locB<-getBeanSub(df_loc)
      #  df_name<-"fungi_family_soil"
      evenS<-function(df){apply(df,1,function(col) diversity(col)/
log(specnumber(col)))}
      dat.indices<-data.frame(Diversity_sh=apply(df_locB,1,function(x)
diversity(x, index = "shannon")),
                              Diversity_si=apply(df_locB,1,function(x)
diversity(x, index = "simpson")),
                              Evenness=apply(df_locB,1,function(x)
diversity(x)/log(specnumber(x))),
                              Richness_chao1=apply(df_locB,
1,function(x) estimateR(round(x), index="chao"))[2,]
      )
      df_annot<-merge(dat.indices,sample_metadata,by="row.names")
      df_annot<-droplevels(df_annot)
      ## plot
      ## melt and add sample metadata
      #
      # renaming Fungi level to metarhizium
      levels(df_annot$Fungi)<-c("M+","M-")
      levels(df_annot$Location)<-c("Root","Rhizospheric Soil")
      #methods t.test wilcox.test kruskal.test anova
      ggplot(df_annot,aes_string(x="Fungi",y=index,Fill="Fungi"))+
        geom_boxplot(alpha=0.8)+facet_wrap(~Insect,scales="free")+
        geom_point(aes(fill=Fungi),size = 3, shape = 21)+
        stat_compare_means(label="p.format",method="wilcox.test")+
        guides(fill=guide_legend("M. robertsii")) +
        #ggtitle(df_name)+
        ylab(paste(index))+
        xlab("M. robertsii")+
        #    scale_x_discrete(labels= c("M+","M-","soil alone"))+
        theme(plot.title = element_text(size = 18, face = "bold"))+
```

```
        theme(axis.text=element_text(size=14),
              axis.title=element_text(size=14)) +
        theme(legend.text=element_text(size=14),
              legend.title=element_text(size=14)) +
        theme(strip.text.x = element_text(size = 14))

  })
  #
  output$divplotS<-renderPlot({
    req(is.null(input$otu)==F)
    df<-abundance_tables_tR[[datasetNameDiv()]]
    index<-indexInput()
    ## Root first
    df_loc<-getSRSub(df,"S")
    #df_locB<-getBeanSub(df_loc)
    #  df_name<-"fungi_family_soil"
    evenS<-function(df){apply(df,1,function(col) diversity(col)/
log(specnumber(col)))}
    dat.indices<-data.frame(Diversity_sh=apply(df_loc,1,function(x)
diversity(x, index = "shannon")),
                            Diversity_si=apply(df_loc,1,function(x)
diversity(x, index = "simpson")),
                            Evenness=apply(df_loc,1,function(x)
diversity(x)/log(specnumber(x))),
                            Richness_chao1=apply(df_loc,1,function(x)
estimateR(round(x), index="chao"))[2,]
    )
    df_annot<-merge(dat.indices,sample_metadata,by="row.names")
    # for the insect subset (no "bulk soil")
    df_annotI<-subset(df_annot,Insect=="Insect")
    # renaming Fungi level to metarhizium
    levels(df_annotI$Fungi)<-c("M+","M-")
    levels(df_annotI$Location)<-c("Root","Rhizospheric Soil")
    df_annotI<-droplevels(df_annotI)
    #methods t.test wilcox.test kruskal.test anova
    plotinsect<-
ggplot(df_annotI,aes_string(x="Fungi",y=index,Fill="Fungi"))+
      geom_boxplot(alpha=0.8)+
      geom_point(aes(fill=Fungi),size = 3, shape = 21)+
      stat_compare_means(label="p.format",method="wilcox.test")+
      guides(fill=guide_legend("M. robertsii")) +
      ggtitle("Insect")+
      ylab(paste(index))+
      xlab("M. robertsii")+
      #scale_x_discrete(labels= c("M+","M-","soil alone"))+
      theme(plot.title = element_text(size = 18, face = "bold"))+
      theme(axis.text=element_text(size=14),
            axis.title=element_text(size=14)) +
      theme(legend.text=element_text(size=14),
            legend.title=element_text(size=14)) +
```

```
      theme(strip.text.x = element_text(size = 14))
    ####
    # for the No- insect subset (no "bulk soil")
    df_annotNI<-subset(df_annot,Insect=="NI")
    # renaming Fungi level to metarhizium
    df_annotNI$Fungi<-as.character(df_annotNI$Fungi)
    df_annotNI$Fungi[which(df_annotNI$Bean =="No bean")]<-"bulk_soil"
    df_annotNI$Fungi <- factor(df_annotNI$Fungi, levels =
c("Fungi","NF","bulk_soil"))
    #df_annotNI$Fungi<-as.factor(df_annotNI$Fungi)
    #revalue(df_annotNI$Fungi, c("Fungi"="M+",
"NF"="M-","bulk_soil=bulk_soil"))
    levels(df_annotNI$Fungi)<-c("M+","M-","bulk_soil")
    levels(df_annotNI$Location)<-c("Root","Rhizospheric Soil")
    #df_annotNI<-droplevels(df_annotNI)
    #methods t.test wilcox.test kruskal.test anova
    my_comparisons <- list( c("M+", "M-"), c("M+", "bulk_soil"),
c("M-", "bulk_soil") )
    plotNinsect<-
ggplot(df_annotNI,aes_string(x="Fungi",y=index,Fill="Fungi"))+
      geom_boxplot(alpha=0.8)+
      geom_point(aes(fill=Fungi),size = 3, shape = 21)+
      stat_compare_means(comparisons=my_comparisons)+
      #stat_compare_means(label="p.format",method="wilcox.test")+
      guides(fill=guide_legend("M. robertsii")) +
      ggtitle("No_insect")+
      ylab(paste(index))+
      xlab("M. robertsii")+
      scale_x_discrete(labels= c("M+","M-","bulk soil"))+
      theme(plot.title = element_text(size = 18, face = "bold"))+
      theme(axis.text=element_text(size=14),
            axis.title=element_text(size=14)) +
      theme(legend.text=element_text(size=14),
            legend.title=element_text(size=14)) +
      theme(strip.text.x = element_text(size = 14))
    grid.arrange(plotinsect, plotNinsect, ncol=2)

  })

  ## now make glm table
  ## all factors otu
  output$table1 <- renderTable({
    req(is.null(input$otu)==F)
    aldex_tab<-aldex_tables[[datasetName0()]]
    #df<-datasetInput()
    otu<-otuInput()
    print(paste("otu selected:",otu))
#
    otu_aldex_results<-aldex_tab[otu,]
    #
```

```
    PvalueColumns <- function(df){
      intersect(
        grep("model" ,colnames(df)),
        grep( "Pr",colnames(df))
      )}
    PvalCols<-PvalueColumns(otu_aldex_results)
    otu_aldex_results_Pvals<-otu_aldex_results[,PvalCols]
    otu_aldex_results_Pvals<-otu_aldex_results_Pvals[,c(1,2,3)]
    otu_aldex_results_Pvals<-signif(otu_aldex_results_Pvals,digits=3)
    out<-
data.frame(Factor=c("M.robertii","Insect","M.robertii*Insect"),Padj=t(
otu_aldex_results_Pvals))
    colnames(out)<-c("Factor","Padj")
    # make nice column names
    return(out)
  },bordered=T)
  ## now glm just for soil by otu
  output$tableS <- renderTable({
    req(is.null(input$otu)==F)
    aldex_tab<-aldex_tables_S[[datasetNameO()]]
    #df<-datasetInput()
    otu<-otuInput()
    print(paste("otu selected:",otu))
    otu_aldex_results<-aldex_tab[otu,]
    #
    PvalueColumns <- function(df){
      intersect(
        grep("model" ,colnames(df)),
        grep( "Pr",colnames(df))
      )}
    PvalCols<-PvalueColumns(otu_aldex_results)
    otu_aldex_results_Pvals<-otu_aldex_results[,PvalCols]
    otu_aldex_results_Pvals<-otu_aldex_results_Pvals[,c(1,2,3)]
    otu_aldex_results_Pvals<-signif(otu_aldex_results_Pvals,digits=3)
    out<-
data.frame(Factor=c("M.robertii","Insect","M.robertii*Insect"),Padj=t(
otu_aldex_results_Pvals))
    colnames(out)<-c("Factor","Padj")
    # make nice column names
    return(out)
  },bordered=T)
  ## now just for root otu
  output$tableR <- renderTable({
    req(is.null(input$otu)==F)
    aldex_tab<-aldex_tables_R[[datasetNameO()]]
    #df<-datasetInput()
    otu<-otuInput()
    print(paste("otu selected:",otu))
    ## melt and add sample metadata
    otu_aldex_results<-aldex_tab[otu,]
```

```
    #head(x.FBS.mm.int.glm[,sort(x.FBS.mm.int.glm[,8])])
    #
    PvalueColumns <- function(df){
      intersect(
        grep("model" ,colnames(df)),
        grep( "Pr",colnames(df))
      )}
    PvalCols<-PvalueColumns(otu_aldex_results)
    otu_aldex_results_Pvals<-otu_aldex_results[,PvalCols]
    otu_aldex_results_Pvals<-otu_aldex_results_Pvals[,c(1,2,3)]
    otu_aldex_results_Pvals<-signif(otu_aldex_results_Pvals,digits=3)
    out<-
data.frame(Factor=c("M.robertii","Insect","M.robertii*Insect"),Padj=t(
otu_aldex_results_Pvals))
    colnames(out)<-c("Factor","Padj")
    # make nice column names
    return(out)
  },bordered=T)
  ## ttest for otu
  ## t-test table all tests
  output$tablet <- renderTable({
    req(is.null(input$otu)==F)
    tt_tab<-tt_tables[[datasetNameO()]]
    #df<-datasetInput()
    otu<-otuInput()
    print(paste("otu selected:",otu))
    #
    tt_tabFI<-tt_tab[["FI"]][otu,]
    tt_tabFnI<-tt_tab[["FnI"]][otu,]
    tt_tabIF<-tt_tab[["IF"]][otu,]
    tt_tabInF<-tt_tab[["InF"]][otu,]
    ##
    result<-
rbind(tt_tabFI[,c(6,8,10)],tt_tabFnI[,c(6,8,10)],tt_tabIF[,c(6,8,10)],
tt_tabInF[,c(6,8,10)])
    colnames(result)<-c("effect_size","welchs Padj","wilcox Padj")
    # I did t-test formula in reverse - I would likeM+/M- to be +ve if
M+ is higher
    result$effect_size<-result$effect_size*-1
    result<-signif(result,digits=3)
    result$Comparison<-c("M+I+ vs M-I+","M+I- vs M-I-","I+M+ vs I-
M+","I+M- vs I-M-")
    # make nice column names
    return(result)
  },bordered=T)
  ## t-test just soil
  output$tabletS <- renderTable({
    req(is.null(input$otu)==F)
    tt_tab<-tt_tables_S[[datasetNameO()]]
    #df<-datasetInput()
```

```
    otu<-otuInput()
    print(paste("otu selected:",otu))
    #
    tt_tabFI<-tt_tab[["FI"]][otu,]
    tt_tabFnI<-tt_tab[["FnI"]][otu,]
    tt_tabIF<-tt_tab[["IF"]][otu,]
    tt_tabInF<-tt_tab[["InF"]][otu,]
    ##
    result<-
rbind(tt_tabFI[,c(6,8,10)],tt_tabFnI[,c(6,8,10)],tt_tabIF[,c(6,8,10)],
tt_tabInF[,c(6,8,10)])
    colnames(result)<-c("effect_size","welchs Padj","wilcox Padj")
    # I did t-test formula in reverse - I would likeM+/M- to be +ve if
M+ is higher
    result$effect_size<-result$effect_size*-1
    result<-signif(result,digits=3)
    result$Comparison<-c("M+I+ vs M-I+","M+I- vs M-I-","I+M+ vs I-
M+","I+M- vs I-M-")
    # make nice column names
    return(result)
  },bordered=T)
  ## t-test just soil
  output$tabletR <- renderTable({
    req(is.null(input$otu)==F)
    tt_tab<-tt_tables_R[[datasetNameO()]]
    #df<-datasetInput()
    otu<-otuInput()
    print(paste("otu selected:",otu))
    #
    tt_tabFI<-tt_tab[["FI"]][otu,]
    tt_tabFnI<-tt_tab[["FnI"]][otu,]
    tt_tabIF<-tt_tab[["IF"]][otu,]
    tt_tabInF<-tt_tab[["InF"]][otu,]
    ##
    result<-
rbind(tt_tabFI[,c(6,8,10)],tt_tabFnI[,c(6,8,10)],tt_tabIF[,c(6,8,10)],
tt_tabInF[,c(6,8,10)])
    colnames(result)<-c("effect_size","welchs Padj","wilcox Padj")
    result<-signif(result,digits=3)
    # I did t-test formula in reverse - I would likeM+/M- to be +ve if
M+ is higher
    result$effect_size<-result$effect_size*-1
    result$Comparison<-c("M+I+ vs M-I+","M+I- vs M-I-","I+M+ vs I-
M+","I+M- vs I-M-")
    # make nice column names
    return(result)
  },bordered=T)
 #### now output for glm tab
  output$glm_tab2 <- renderTable({
#    req(is.null(comparisonInput())==F)
```

```
   # df<-datasetInput()
    aldex_tab<-aldex_tables[[datasetNameG()]]
    pval<-pvalInputG()
    compFacts<-FactorsInput()
    ## select based on Factor of interest
     if(compFacts=="M.robertii"){
      aldex_tab.cF.sig<- aldex_tab[aldex_tab[,8]<pval,]
      aldex_tab.cF.sig<-aldex_tab.cF.sig[,c(5,8)]
      colnames(aldex_tab.cF.sig)<-c("coefficient","M.robertii_Padj")
     }
    if(compFacts=="Insect"){
      aldex_tab.cF.sig<- aldex_tab[aldex_tab[,12]<pval,]
      aldex_tab.cF.sig<-aldex_tab.cF.sig[,c(9,12)]
      colnames(aldex_tab.cF.sig)<-c("coefficient","Insect_Padj")
    }
    if(compFacts=="M.robertii*Insect"){
      aldex_tab.cF.sig<- aldex_tab[aldex_tab[,16]<pval,]
      aldex_tab.cF.sig<-aldex_tab.cF.sig[,c(13,16)]
      colnames(aldex_tab.cF.sig)<-
c("coefficient","M.robertii*Insect_Padj")
    }
#
    aldex_tab.cF.sig<-signif(aldex_tab.cF.sig,digits=3)
    aldex_tab.cF.sig<-aldex_tab.cF.sig[order(aldex_tab.cF.sig[,1]),]
    aldex_tab.cF.sig$OTU<-rownames(aldex_tab.cF.sig)
    return(aldex_tab.cF.sig)
  },striped = TRUE, bordered = TRUE)
  ## now just for soil
  output$glm_tabS <- renderTable({
    #    req(is.null(comparisonInput())==F)
    # df<-datasetInput()
    aldex_tab<-aldex_tables_S[[datasetNameG()]]
    pval<-pvalInputG()
    compFacts<-FactorsInput()
    ## select based on Factor of interest
    if(compFacts=="M.robertii"){
      aldex_tab.cF.sig<- aldex_tab[aldex_tab[,8]<pval,]
      aldex_tab.cF.sig<-aldex_tab.cF.sig[,c(5,8)]
      colnames(aldex_tab.cF.sig)<-c("coefficient","M.robertii_Padj")
    }
    if(compFacts=="Insect"){
      aldex_tab.cF.sig<- aldex_tab[aldex_tab[,12]<pval,]
      aldex_tab.cF.sig<-aldex_tab.cF.sig[,c(9,12)]
      colnames(aldex_tab.cF.sig)<-c("coefficient","Insect_Padj")
    }
    if(compFacts=="M.robertii*Insect"){
      aldex_tab.cF.sig<- aldex_tab[aldex_tab[,16]<pval,]
      aldex_tab.cF.sig<-aldex_tab.cF.sig[,c(13,16)]
      colnames(aldex_tab.cF.sig)<-
c("coefficient","M.robertii*Insect_Padj")
```

```
    }
    #
    aldex_tab.cF.sig<-signif(aldex_tab.cF.sig,digits=3)
    aldex_tab.cF.sig<-aldex_tab.cF.sig[order(aldex_tab.cF.sig[,1]),]
    aldex_tab.cF.sig$OTU<-rownames(aldex_tab.cF.sig)
    return(aldex_tab.cF.sig)
  },striped = TRUE, bordered = TRUE)
  ## now just for root
  output$glm_tabR <- renderTable({
    #    req(is.null(comparisonInput())==F)
    # df<-datasetInput()
    aldex_tab<-aldex_tables_R[[datasetNameG()]]
    pval<-pvalInputG()
    compFacts<-FactorsInput()
    ## select based on Factor of interest
    if(compFacts=="M.robertii"){
      aldex_tab.cF.sig<- aldex_tab[aldex_tab[,8]<pval,]
      aldex_tab.cF.sig<-aldex_tab.cF.sig[,c(5,8)]
      colnames(aldex_tab.cF.sig)<-c("coefficient","M.robertii_Padj")
    }
    if(compFacts=="Insect"){
      aldex_tab.cF.sig<- aldex_tab[aldex_tab[,12]<pval,]
      aldex_tab.cF.sig<-aldex_tab.cF.sig[,c(9,12)]
      colnames(aldex_tab.cF.sig)<-c("coefficient","Insect_Padj")
    }
    if(compFacts=="M.robertii*Insect"){
      aldex_tab.cF.sig<- aldex_tab[aldex_tab[,16]<pval,]
      aldex_tab.cF.sig<-aldex_tab.cF.sig[,c(13,16)]
      colnames(aldex_tab.cF.sig)<-
c("coefficient","M.robertii*Insect_Padj")
    }
    #
    aldex_tab.cF.sig<-signif(aldex_tab.cF.sig,digits=3)
    aldex_tab.cF.sig<-aldex_tab.cF.sig[order(aldex_tab.cF.sig[,1]),]
    aldex_tab.cF.sig$OTU<-rownames(aldex_tab.cF.sig)
    return(aldex_tab.cF.sig)
  },striped = TRUE, bordered = TRUE)
  ### output for ttest tab t-test
  output$tt_tab2 <- renderTable({
    #    req(is.null(comparisonInput())==F)
    # df<-datasetInput()
    tt_tab<-tt_tables[[datasetNameT()]]
    pval<-pvalInputT()
    compFacts<-comparisonInput()
    ## select based on Factor of interest
    if(compFacts=="M+I+ vs M-I+"){
      tt_tab.comp<- tt_tab[["FI"]]
    }
    if(compFacts=="M+I- vs M-I-"){
      tt_tab.comp<- tt_tab[["FnI"]]
```

```
    }
    if(compFacts=="I+M+ vs I-M+"){
      tt_tab.comp<- tt_tab[["IF"]]
    }
    if(compFacts=="I+M- vs I-M-"){
      tt_tab.comp<- tt_tab[["InF"]]
    }
    #
    tt_tab.comp.sig<-subset(tt_tab.comp,we.ep<=pval|wi.ep<=pval)
    tt_tab.comp.sig<-tt_tab.comp.sig[,c(6,8,10)]
    colnames(tt_tab.comp.sig)<-c("effect_size","welchs padj","wilcox
padj")
    tt_tab.comp.sig$OTU<-rownames(tt_tab.comp.sig)
    # I did t-test formula in reverse - I would likeM+/M- to be +ve if
M+ is higher
    tt_tab.comp.sig$effect_size<-tt_tab.comp.sig$effect_size*-1
    return(tt_tab.comp.sig)
  },striped = TRUE, bordered = TRUE)
  #
  output$tt_tabR <- renderTable({
    #    req(is.null(comparisonInput())==F)
    # df<-datasetInput()
    tt_tab<-tt_tables_R[[datasetNameT()]]
    pval<-pvalInputT()
    compFacts<-comparisonInput()
    ## select based on Factor of interest
    if(compFacts=="M+I+ vs M-I+"){
      tt_tab.comp<- tt_tab[["FI"]]
    }
    if(compFacts=="M+I- vs M-I-"){
      tt_tab.comp<- tt_tab[["FnI"]]
    }
    if(compFacts=="I+M+ vs I-M+"){
      tt_tab.comp<- tt_tab[["IF"]]
    }
    if(compFacts=="I+M- vs I-M-"){
      tt_tab.comp<- tt_tab[["InF"]]
    }
    #
    tt_tab.comp.sig<-subset(tt_tab.comp,we.ep<=pval|wi.ep<=pval)
    tt_tab.comp.sig<-tt_tab.comp.sig[,c(6,8,10)]
    colnames(tt_tab.comp.sig)<-c("effect_size","welchs padj","wilcox
padj")
    tt_tab.comp.sig$OTU<-rownames(tt_tab.comp.sig)
    # I did t-test formula in reverse - I would likeM+/M- to be +ve if
M+ is higher
    tt_tab.comp.sig$effect_size<-tt_tab.comp.sig$effect_size*-1
    return(tt_tab.comp.sig)
  },striped = TRUE, bordered = TRUE)
  #
```

```
  output$tt_tabS <- renderTable({
    #    req(is.null(comparisonInput())==F)
    # df<-datasetInput()
    tt_tab<-tt_tables_S[[datasetNameT()]]
    pval<-pvalInputT()
    compFacts<-comparisonInput()
    ## select based on Factor of interest
    if(compFacts=="M+I+ vs M-I+"){
      tt_tab.comp<- tt_tab[["FI"]]
    }
    if(compFacts=="M+I- vs M-I-"){
      tt_tab.comp<- tt_tab[["FnI"]]
    }
    if(compFacts=="I+M+ vs I-M+"){
      tt_tab.comp<- tt_tab[["IF"]]
    }
    if(compFacts=="I+M- vs I-M-"){
      tt_tab.comp<- tt_tab[["InF"]]
    }
    #
    tt_tab.comp.sig<-subset(tt_tab.comp,we.ep<=pval|wi.ep<=pval)
    tt_tab.comp.sig<-tt_tab.comp.sig[,c(6,8,10)]
    colnames(tt_tab.comp.sig)<-c("effect_size","welchs padj","wilcox
padj")
    tt_tab.comp.sig$OTU<-rownames(tt_tab.comp.sig)
    # I did t-test formula in reverse - I would likeM+/M- to be +ve if
M+ is higher
    tt_tab.comp.sig$effect_size<-tt_tab.comp.sig$effect_size*-1
    return(tt_tab.comp.sig)
  },striped = TRUE, bordered = TRUE)
  ##
  #### downloadData ##
  # Downloadable csv of selected dataset ----
  output$downloadData <- downloadHandler(
    filename = function() {
      paste("metarhiz-microbiome",datasetNameD(), ".csv", sep = "")
    },
    content = function(file) {
      write.csv(abundance_tables_tR[[datasetNameD()]], file, row.names
= TRUE)
    }
  )
    output$downloadShiny <- downloadHandler(
      filename="metarhiz-microbiome_app.R",
      content <- function(file) {
        file.copy("app.R", file)
      }
  )
    output$downloadPreanal <- downloadHandler(
      filename="metarhiz-microbiome_preanalysis.R",
```

```
      content <- function(file) {
        file.copy("app_ALDEx2_mvabund_shiny_preanalysisv2.R", file)
      }
    )
}
  ### end of server
shinyApp(ui=ui,server=server)
```
