# Supplementary material for: Plant microbiome analysis after Metarhizium amendment reveals increases in abundance of plant growth-promoting organisms and maintenance of disease-suppressive soil
Source: PLoS One. 2020 Apr 10;15(4):e0231150. doi: 10.1371/journal.pone.0231150 (PMC7147777; doi:10.1371/journal.pone.0231150)
Supplement: S1 Table — (PDF) [file pone.0231150.s004.pdf]

**S1 Table. Experimental treatment setup for microbiome analysis.**

| Treatment | Bean | *Metarhizium* | Insect | Total Pots (pooled samples) | Total Root Samples | Total Soil Samples |
|---|---|---|---|---|---|---|
| M+I+ | + | + | + | 9 (3) | 3 | 3 |
| M+I- | + | + | - | 9 (3) | 3 | 3 |
| M-I+ | + | - | + | 9 (3) | 3 | 3 |
| M-I- | + | - | - | 9 (3) | 3 | 3 |
| Soil | - | - | - | 9 (3) | N/A | 3 |
