# Supplementary material for: Plant microbiome analysis after Metarhizium amendment reveals increases in abundance of plant growth-promoting organisms and maintenance of disease-suppressive soil
Source: PLoS One. 2020 Apr 10;15(4):e0231150. doi: 10.1371/journal.pone.0231150 (PMC7147777; doi:10.1371/journal.pone.0231150)
Supplement: S2 Table — (PDF) [file pone.0231150.s005.pdf]

S2 Table. OTUs enriched in root and rhizosphere soil.

Target	Location	Effect	Adj-P	OTU	Taxonomic classification
Fungi	Rhizosphere	3.43	<0.001	Otu0004	s__Hygrocybe_sp
	Soil	2.97	<0.001	Otu0029	k__Fungi_un.
		2.54	<0.001	Otu0030	k__Fungi_un.
		2.50	<0.001	Otu0027	k__Fungi_un.
		2.39	<0.001	Otu0015	c__Eurotiomycetes_un.
		2.39	<0.001	Otu0021	s__Spizellomyces_sp
		2.39	<0.001	Otu0020	o__Agaricales_un.
		2.22	0.001	Otu0028	s__Staphylotrichum_coccosporum
		2.17	<0.001	Otu0074	k__Fungi_un.
		2.00	<0.001	Otu0036	p__Ascomycota_un.
		1.80	0.001	Otu0040	s__Spizellomyces_sp
		1.79	<0.001	Otu0052	k__Fungi_un.
		1.76	0.002	Otu0104	p__Basidiomycota_un.
		1.76	<0.001	Otu0075	s__Hygrocybe_glutinipes
		1.63	0.002	Otu0116	k__Fungi_un.
		1.60	0.002	Otu0091	s__Orbiliomycetes_sp
		1.54	0.002	Otu0114	c__Sordariomycetes_un.
		1.50	0.001	Otu0047	k__Fungi_un.
		1.46	0.002	Otu0106	k__Fungi_un.
		1.45	0.008	Otu0082	c__Agaricomycetes_un.
		1.40	0.006	Otu0022	g__Mortierella_un.
		1.38	0.004	Otu0129	k__Fungi_un.
		1.37	0.004	Otu0152	k__Fungi_un.
		1.34	0.006	Otu0205	k__Fungi_un.
		1.31	0.006	Otu0220	k__Fungi_un.
		1.29	0.013	Otu0006	g__Mortierella_un.
		1.28	0.002	Otu0043	s__Trichoglossum_sp
		1.27	0.010	Otu0123	k__Fungi_un.
		1.26	0.003	Otu0065	f__Ustilaginaceae_un.
		1.25	0.006	Otu0230	k__Fungi_un.
		1.24	0.004	Otu0101	k__Fungi_un.
		1.24	0.006	Otu0174	k__Fungi_un.
		1.20	0.005	Otu0133	k__Fungi_un.
		1.19	0.010	Otu0194	k__Fungi_un.
		1.17	0.018	Otu0025	f__Chaetomiaceae_un.
		1.17	0.009	Otu0229	k__Fungi_un.
		1.17	0.007	Otu0080	p__Ascomycota_un.
		1.14	0.017	Otu0084	p__Ascomycota_un.
		1.13	0.011	Otu0056	c__Eurotiomycetes_un.
		1.12	0.007	Otu0060	g__Staphylotrichum_un.
		1.11	0.005	Otu0035	k__Fungi_un.
		1.07	0.037	Otu0012	s__Mortierella_exigua
		1.05	0.011	Otu0203	p__Ascomycota_un.
		1.04	0.031	Otu0071	s__Eurotiomycetes_sp

Target	Location	Effect	Adj-P	OTU	Taxonomic classification
Fungi	Rhizosphere	1.04	0.029	Otu0090	s__Geoglossales_sp
	Soil	1.01	0.034	Otu0177	p__Ascomycota_un.
Fungi	Root	-1.00	0.014	Otu0350	s__Ceratobasidiaceae_sp
		-1.01	0.022	Otu0332	p__Ascomycota_un.
		-1.06	0.020	Otu0270	o__Agaricales_un.
		-1.13	0.007	Otu0111	s__Chaetothyriaceae_sp
		-1.18	0.017	Otu0377	s__Trichoderma_longibrachiatum
		-1.21	0.013	Otu0153	s__Laetisaria_arvalis
		-1.22	0.005	Otu0086	s__Marasmius_curreyi
		-1.24	0.007	Otu0240	s__Acremonium_stromaticum
		-1.26	0.005	Otu0273	f__Helotiales_fam_Incertae_sedis_un.
		-1.31	0.005	Otu0416	s__Ophiosphaerella_korrae
		-1.42	0.016	Otu0050	s__Gibberella_tricincta
		-1.46	0.005	Otu0584	s__Metarhizium_marquandii
		-1.48	0.002	Otu0241	s__Flagelloscypha_minutissima
		-1.52	0.002	Otu0368	k__Fungi_un.
		-1.56	0.008	Otu0275	s__Codinaea_acaciae
		-1.56	0.006	Otu0108	s__Gibberella_intricans
		-1.59	0.002	Otu0199	g__Periconia_un.
		-1.62	0.004	Otu0122	s__Xylariales_sp
		-1.67	0.008	Otu0151	g__Fusarium_un.
		-1.67	0.003	Otu0443	k__Fungi_un.
		-1.72	0.008	Otu0057	s__Paraphoma_chrysanthemicola
		-1.79	0.001	Otu0250	s__Roussoella_hysterioides
		-1.98	<0.001	Otu0209	s__Ilyonectria_robusta
		-2.00	<0.001	Otu0202	k__Fungi_un.
		-2.03	0.002	Otu0163	k__Fungi_un.
		-2.06	0.023	Otu0081	s__Gibellulopsis_nigrescens
		-2.13	<0.001	Otu0544	g__Fusarium_un.
		-2.21	<0.001	Otu0014	o__Pleosporales_un.
		-2.22	0.008	Otu0164	s__Cylindrocarpon_sp
		-2.53	0.001	Otu0373	p__Ascomycota_un.
		-2.65	0.036	Otu0010	s__Fusarium_solani
		-2.66	<0.001	Otu0083	s__Dactylonectria_anthuriicola
		-2.94	<0.001	Otu0165	g__Fusarium_un.
		-2.96	0.007	Otu0058	o__Xylariales_un.
		-3.08	0.003	Otu0062	s__Ilyonectria_macrodidyma
		-3.12	<0.001	Otu0185	s__Fusarium_oxysporum
		-3.30	<0.001	Otu0046	k__Fungi_un.
		-3.36	0.033	Otu0039	s__Fusarium_solani
		-3.97	0.035	Otu0005	s__Clonostachys_rosea
		-4.61	0.013	Otu0011	f__Helotiales_fam_Incertae_sedis_un.
		-6.16	0.024	Otu0001	k__Fungi_un.
		-6.94	0.001	Otu0002	s__Fusarium_oxysporum

Target	Location	Effect	Adj-P	OTU	Taxonomic classification
Bacteria	Rhizosphere	1.96	<0.001	Otu00002	o__iii1-15_un.
	Soil	1.21	0.010	Otu00027	f__Ellin5301_un.
		1.20	0.011	Otu00024	o__Myxococcales_un.
		1.19	0.008	Otu00070	f__0319-6A21_un.
		1.19	0.014	Otu00013	o__SC-I-84_un.
		1.15	0.011	Otu00062	f__0319-6A21_un.
		1.09	0.013	Otu00056	f__C111_un.
		1.07	0.012	Otu00007	o__iii1-15_un.
		1.05	0.022	Otu00072	o__SC-I-84_un.
Bacteria	Root	-1.06	0.012	Otu00415	g__Dyadobacter_un.
		-1.10	0.015	Otu00102	s__Serratia_marcescens
		-1.22	0.006	Otu00182	g__Olivibacter_un.
		-1.23	0.020	Otu00544	f__Cytophagaceae_un.
		-1.24	0.008	Otu00111	g__Pedobacter_un.
		-1.31	0.003	Otu00011	g__DA101_un.
		-1.32	0.004	Otu00484	f__Chitinophagaceae_un.
		-1.35	0.005	Otu00594	f__Chitinophagaceae_un.
		-1.36	0.011	Otu00411	s__Microbacterium_chocolatum
		-1.37	0.014	Otu00082	g__Pedobacter_un.
		-1.38	0.003	Otu00064	g__Lysobacter_un.
		-1.40	0.012	Otu00552	s__Bosea_genosp.
		-1.46	0.002	Otu00257	g__Dyadobacter_un.
		-1.46	<0.001	Otu00012	s__Flavobacterium_succinicans
		-1.55	0.015	Otu00455	f__Micromonosporaceae_un.
		-1.55	0.004	Otu00395	g__Aminobacter_un.
		-1.55	0.002	Otu00260	s__Flavobacterium_succinicans
		-1.60	0.006	Otu00310	f__Sphingobacteriaceae_un.
		-1.61	0.002	Otu00261	f__Sphingomonadaceae_un.
		-1.62	<0.001	Otu00053	g__Chryseobacterium_un.
		-1.63	<0.001	Otu00126	s__Arthrobacter_psychrolactophilus
		-1.68	<0.001	Otu00001	g__DA101_un.
		-1.70	0.001	Otu00569	s__Duganella_nigrescens
		-1.73	0.002	Otu00568	f__Chitinophagaceae_un.
		-1.74	<0.001	Otu00289	f__Oxalobacteraceae_un.
		-1.78	0.001	Otu00550	g__Chitinophaga_un.
		-1.80	<0.001	Otu00158	g__Agrobacterium_un.
		-1.83	<0.001	Otu00117	s__Chitinophaga_arvensicola
		-1.85	<0.001	Otu00041	f__Enterobacteriaceae_un.
		-1.86	0.002	Otu00483	g__Pedobacter_un.
		-1.88	<0.001	Otu00474	f__Chitinophagaceae_un.
		-1.95	<0.001	Otu00486	f__Chitinophagaceae_un.
		-1.95	<0.001	Otu00236	s__Luteibacter_rhizovicinus
		-1.99	<0.001	Otu00133	g__Kribbella_un.
		-2.12	<0.001	Otu00457	g__Promicromonospora_un.

Target	Location	Effect	Adj-P	OTU	Taxonomic classification
Bacteria	Root	-2.13	<0.001	Otu00078	g__Ochrobactrum_un.
		-2.15	<0.001	Otu00383	f__Cytophagaceae_un.
		-2.20	0.001	Otu00197	g__Achromobacter_un.
		-2.21	<0.001	Otu00206	g__Labrys_un.
		-2.22	<0.001	Otu00025	f__Comamonadaceae_un.
		-2.23	<0.001	Otu00386	k__Bacteria_un.
		-2.23	<0.001	Otu00138	s__Acinetobacter_rhizosphaerae
		-2.32	<0.001	Otu00085	s__Bacillus_flexus
		-2.43	<0.001	Otu00431	g__Dyadobacter_un.
		-2.46	<0.001	Otu00370	g__Niastella_un.
		-2.49	<0.001	Otu00141	g__Dyadobacter_un.
		-2.62	<0.001	Otu00513	o__Actinomycetales_un.
		-2.64	<0.001	Otu00337	o__Actinomycetales_un.
		-2.64	<0.001	Otu00460	s__Duganella_nigrescens
		-2.65	<0.001	Otu00029	g__Yersinia_un.
		-2.65	<0.001	Otu00023	g__Novosphingobium_un.
		-2.69	<0.001	Otu00080	g__Mycobacterium_un.
		-2.74	<0.001	Otu00054	g__Sphingomonas_un.
		-2.79	<0.001	Otu00336	g__Streptomyces_un.
		-2.79	<0.001	Otu00156	s__Burkholderia_bryophila
		-2.80	<0.001	Otu00280	f__Actinosynnemataceae_un.
		-2.81	<0.001	Otu00017	g__Ochrobactrum_un.
		-2.82	<0.001	Otu00305	f__Sphingobacteriaceae_un.
		-2.95	<0.001	Otu00442	o__Rhizobiales_un.
		-2.96	<0.001	Otu00229	s__Glycomyces_harbinensis
		-3.00	<0.001	Otu00036	s__Stenotrophomonas_retroflexus
		-3.13	<0.001	Otu00003	s__Sphingobacterium_multivorum
		-3.23	<0.001	Otu00096	f__Sphingobacteriaceae_un.
		-3.27	<0.001	Otu00060	f__Sphingobacteriaceae_un.
		-3.37	<0.001	Otu00108	g__Arthrobacter_un.
		-3.39	<0.001	Otu00168	g__Chitinophaga_un.
		-3.61	<0.001	Otu00149	g__Mesorhizobium_un.
		-3.64	<0.001	Otu00219	f__Sphingobacteriaceae_un.
		-3.99	<0.001	Otu00039	g__Pseudomonas_un.
		-4.01	<0.001	Otu00015	f__Comamonadaceae_un.
		-4.17	<0.001	Otu00032	s__Methylotenera_mobilis
		-4.18	<0.001	Otu00129	g__Dyadobacter_un.
		-4.20	<0.001	Otu00218	g__Amycolatopsis_un.
		-4.33	<0.001	Otu00083	s__Caulobacter_henricii
		-4.93	<0.001	Otu00066	f__Comamonadaceae_un.
		-4.98	<0.001	Otu00016	g__Bradyrhizobium_un.
		-5.13	<0.001	Otu00018	g__Agrobacterium_un.
		-5.30	<0.001	Otu00042	g__Streptomyces_un.
		-5.48	<0.001	Otu00009	f__Oxalobacteraceae_un.
		-6.23	<0.001	Otu00008	g__Streptomyces_un.

Target	Location	Effect	Adj-*P*	OTU	Taxonomic classification
Bacteria	Root	-6.67	<0.001	Otu00010	g__Niastella_un.
		-7.07	<0.001	Otu00005	f__Oxalobacteraceae_un.
		-7.36	<0.001	Otu00021	s__Rhizobium_leguminosarum
		-8.03	<0.001	Otu00004	s__Streptomyces_mirabilis

Note: Enriched OTUs were determined by GLM analysis. A positive effect indicates OTU is enriched in soil, a negative effect indicates OTU is enriched in the root. Adj-*P* = Benjamini-Hochberg adjusted P value; un. = unclassified.
