# Supplementary material for: Plant microbiome analysis after Metarhizium amendment reveals increases in abundance of plant growth-promoting organisms and maintenance of disease-suppressive soil
Source: PLoS One. 2020 Apr 10;15(4):e0231150. doi: 10.1371/journal.pone.0231150 (PMC7147777; doi:10.1371/journal.pone.0231150)
Supplement: S3 Table — (PDF) [file pone.0231150.s006.pdf]

S3 Table. Summary of generalized linear model (GLM) of bacterial taxa significantly affected by treatment.

Factor	Location	Coeff.	adj-P	Taxon
Metarhizium Galleria*	Root	-10.20	0.03	k__Bacteria; p__Chloroflexi; c__TK10; o__AKYG885; f__Dolo_23
		-4.15	0.04	k__Bacteria;p__Proteobacteria;c__Gammaproteobacteria;o__Pseudomonadales;f__Moraxellaceae;g__Acinetobacter
	Soil	8.78	0.01	k__Bacteria; p__Bacteroidetes; c__Sphingobacteriia; o__Sphingobacteriales; f__Sphingobacteriaceae; g__Olivibacter
		10.20	0.01	k__Bacteria; p__Bacteroidetes; c__Sphingobacteriia; o__Sphingobacteriales; f__Sphingobacteriaceae; g__Solitalea
		0.80	0.04	k__Bacteria;p__Planctomycetes;c__Planctomycetia;o__Gemmatales;f__Isosphaeraceae
		2.05	0.02	k__Bacteria; p__Chloroflexi; c__Thermomicrobia; o__JG30-KF-CM45; f_un.
		0.90	0.03	k__Bacteria; p__Actinobacteria; c__Thermoleophilia; o__Solirubrobacterales; f_un.
Metarhizium	Root	3.75	0.01	k__Bacteria;p__Proteobacteria;c__Gammaproteobacteria;o__Pseudomonadales;f__Moraxellaceae;g__Acinetobacter
		5.35	0.02	k__Bacteria; p__Proteobacteria; c__Alphaproteobacteria; o__Rhizobiales; f__Rhizobiaceae; g_un.
		3.89	0.03	k__Bacteria; p__Bacteroidetes; c__Flavobacteriia; o__Flavobacteriales; f__Weeksellaceae; g__Chryseobacterium
		5.02	0.04	k__Bacteria; p__Verrucomicrobia; c__Pedosphaerae; o__Pedosphaerales; f_un.
		2.82	0.04	k__Bacteria; p__Proteobacteria; c__Betaproteobacteria; o_un.
		4.90	0.04	k__Bacteria; p__Actinobacteria; c__Actinobacteria; o__Actinomycetales; f__Microbacteriaceae; g__Microbacterium
		4.70	0.05	k__Bacteria; p__Bacteroidetes; c__Sphingobacteriia; o__Sphingobacteriales; f__Sphingobacteriaceae; g__Sphingobacterium
	Soil	0.75	0.00	k__Bacteria;p__Verrucomicrobia;c__Spartobacteria;o__Chthoniobacterales;f__Chthoniobacteraceae;g__DA101
		0.73	0.01	k__Bacteria;p__Nitrospirae; c__Nitrospira;o__Nitrospirales;f__X0319.6A21; g_un.
		0.70	0.02	k__Bacteria;p__Proteobacteria;c__Alphaproteobacteria;o__Rhodospirillales;f__Rhodospirillaceae; g_un.
		0.67	0.02	k__Bacteria; p__Acidobacteria; c__Chloracidobacteria; o__RB41; f_un.
		0.66	0.02	k__Bacteria;p__Proteobacteria;c__Deltaproteobacteria;o__Syntrophobacterales;f__Syntrophobacteraceae; g_un.
		1.44	0.03	k__Bacteria; p__Nitrospirae; c__Nitrospira; o__Nitrospirales; f__Nitrospiraceae; g__Nitrospira
		0.51	0.03	k__Bacteria; p__Acidobacteria; c__Acidobacteria-6; o__iii1.15; f_un.
		1.00	0.03	k__Bacteria; p__Proteobacteria; c__Gammaproteobacteria; o__Legionellales; f__Coxiellaceae
Galleria	Root	3.76	0.01	k__Bacteria;p__Proteobacteria;c__Gammaproteobacteria;o__Pseudomonadales;f__Moraxellaceae;g__Acinetobacter
		5.33	0.02	k__Bacteria; p__Proteobacteria; c__Alphaproteobacteria; o__Rhizobiales; f__Rhizobiaceae; g_un.
		5.18	0.03	k__Bacteria; p__Verrucomicrobia; c__Pedosphaerae; o__Pedosphaerales; f_un.
		2.32	0.04	k__Bacteria; p__Planctomycetes; c__Planctomycetia; o__Planctomycetales; f__Planctomycetaceae; g__Planctomyces
		6.79	0.05	k__Bacteria;p__Acidobacteria;c__Acidobacteria-6;o__iii1-15;f__RB40
	Soil	-0.91	0.01	k__Bacteria;p__Actinobacteria;c__Thermoleophilia;o__Solirubrobacterales_un.
		-0.76	0.01	k__Bacteria; p__Actinobacteria; c__Thermoleophilia; o__Gaiellales; f__Gaiellaceae
		-0.92	0.01	k__Bacteria; p__Proteobacteria; c__Alphaproteobacteria; o__Rhizobiales; f__Bradyrhizobiaceae; g__Bradyrhizobium
		-0.82	0.02	k__Bacteria;p__Actinobacteria;c__Thermoleophilia;o__Solirubrobacterales; f_Solirubrobacteraceae
		-1.48	0.02	k__Bacteria; p__Chloroflexi; c__Thermomicrobia; o__JG30-KF-CM45; f_un.
		-0.64	0.03	k__Bacteria;p__Planctomycetes;c__Planctomycetia;o__Gemmatales;f__Gemmataceae; g_un.
		-0.68	0.03	k__Bacteria; p__Chloroflexi; c__Ellin6529_un.
		0.49	0.03	k__Bacteria; p__Acidobacteria; c__Chloracidobacteria; o__RB41; f_un.
		-0.69	0.03	k__Bacteria; p__Proteobacteria; c__Alphaproteobacteria; o__Rhizobiales; f__Hyphomicrobiaceae; g__Rhodoplanes
		-.577	0.04	k__Bacteria; p__Bacteroidetes; c__Sphingobacteriia; o__Sphingobacteriales; f__Sphingobacteriaceae; g__Solitalea
		-0.45	0.04	k__Bacteria;p__Actinobacteria;c__Acidimicrobiia;o__Acidimicrobiales; f__C111; g_un.
		-1.62	0.04	k__Bacteria; p__Actinobacteria; c__Actinobacteria; o__Actinomycetales; f__Frankiaceae; g_un.
		1.15	0.05	k__Bacteria; p__Proteobacteria; c__Deltaproteobacteria; o__NB1.j; f__NB1.i

Coeff. = GLM (α = 0.05) coefficient; adj-P = FDR-adjusted P value; un. = unclassified
