# Supplementary material for: Plant microbiome analysis after Metarhizium amendment reveals increases in abundance of plant growth-promoting organisms and maintenance of disease-suppressive soil
Source: PLoS One. 2020 Apr 10;15(4):e0231150. doi: 10.1371/journal.pone.0231150 (PMC7147777; doi:10.1371/journal.pone.0231150)
Supplement: S4 Table — (PDF) [file pone.0231150.s007.pdf]

S4 Table. Summary of generalized linear model (GLM) of fungal taxa significantly affected by treatment.

Factor	Location	Coeff.	adj-P	Taxon
Metarhizium Galleria*	Root	-9.79	0.05	k__Fungi;p__Ascomycota;c__Sordariomycetes;o__Sordariales;f__Chaetomiaceae;g__Chaetomium
	Soil	-3.88	0.00	k__Fungi;p__Ascomycota;c__Sordariomycetes;o__Sordariales;f__Chaetomiaceae;g__Chaetomium
		3.90	0.01	k__Fungi;p__Ascomycota;c__Sordariomycetes;o__Hypocreales;f__Hypocreaceae
		-10.20	0.02	k__Fungi;p__Chytridiomycota;c__Rhizophlyctidomycetes;o__Rhizophlyctidales;f__Rhizophlyctidaceae;g__Rhizophlyctis
		-2.81	0.03	k__Fungi;p__Ascomycota;c__Sordariomycetes;o__Sordariales;f__Chaetomiaceae
		7.82	0.04	k__Fungi;p__Basidiomycota;c__Tremellomycetes;o__Filobasidiales;f__Piskurozymaceae
		-5.61	0.05	k__Fungi;p__Ascomycota;c__Sordariomycetes;o__Hypocreales;f__Hypocreaceae; g_Trichoderma
Metarhizium	Root	3.83	0.00	k__Fungi;p__Basidiomycota;c__Agaricomycetes;o__Russulales;f__Stephanosporaceae
		-4.57	0.04	k__Fungi;p__Basidiomycota;c__Agaricomycetes; f_un.
		-0.95	0.05	k__Fungi;p__Ascomycota;c__Sordariomycetes;o__Hypocreales;f__Nectriaceae; g_Ilyonectria
	Soil	6.27	0.03	k__Fungi;p__Basidiomycota;c__Agaricomycetes;o__Hymenochaetales;f__Schizoporaceae
Galleria	Root	3.33	0.01	k__Fungi;p__Basidiomycota;c__Agaricomycetes;o__Russulales;f__Stephanosporaceae
	Soil	-5.60	0.04	k__Fungi;p__Chytridiomycota; c_Chytridiomycetes; o_un.
		4.08	0.04	k__Fungi;p__Basidiomycota;c__Cystobasidiomycetes;o__Erythrobasidiales;f__Erythrobasidiales_famIncertaesedis;g_Sakaguchia
		-1.90	0.04	k__Fungi;p__Ascomycota;c__Sordariomycetes;o__Chaetosphaeriales;f__Chaetosphaeriaceae
		-5.15	0.03	k__Fungi;p__Ascomycota;c__Dothideomycetes;o__Pleosporales;f__Phaeosphaeriaceae
		7.42	0.03	k__Fungi;p__Zygomycota;c__Mucoromycotina_cls_Incertae_sedis;o__Mucorales;f__Cunninghamellaceae; g__Cunninghamella;s__Cunninghamella_elegans

Coeff. = GLM (α = 0.05) coefficient; Adj-P = FDR-adjusted P value; un. = unclassified
