# Supplementary material for: Plant microbiome analysis after Metarhizium amendment reveals increases in abundance of plant growth-promoting organisms and maintenance of disease-suppressive soil
Source: PLoS One. 2020 Apr 10;15(4):e0231150. doi: 10.1371/journal.pone.0231150 (PMC7147777; doi:10.1371/journal.pone.0231150)
Supplement: S5 Table — (PDF) [file pone.0231150.s008.pdf]

S5 Table. Bacterial taxa significantly affected by *Metarhizium robertsii* amendment determined by Welch's t-test.

Comparison	Location	Effect Size	adj-P	Taxon
**M+** I- vs. M- I-	Soil	-2.98	0.02	k__Bacteria;p__Actinobacteria;c__Acidimicrobiia;o__Acidimicrobiales; f__C111
		-2.10	0.02	k__Bacteria;p__Actinobacteria;c__Thermoleophilia;o__Solirubrobacterales; f_un.
		-2.43	0.03	k__Bacteria;p__Chloroflexi;c__Thermomicrobia;o__JG30-KF-CM45; f_un.
		-2.77	0.03	k__Bacteria;p__Planctomycetes;c__Planctomycetia;o__Gemmatales;f__Gemmataceae
		-2.43	0.03	k__Bacteria;p__Planctomycetes;c__Planctomycetia;o__Gemmatales;f__Isosphaeraceae
		-2.29	0.03	k__Bacteria;p__Verrucomicrobia;c__Spartobacteria;o__Chthoniobacterales;f__Chthoniobacteraceae;g__DA101
		-2.22	0.04	k__Bacteria;p__Actinobacteria;c__MB-A2-108;o__0319-7L14; f_un.
		-2.10	0.04	k__Bacteria;p__Latescibacteria(WS3);c__PRR-12;o__Sediment-1;f__PRR-10
		-1.99	0.04	k__Bacteria;p__Proteobacteria;c__Alphaproteobacteria;o__Rhodospirillales;f__Rhodospirillaceae
		-2.03	0.04	k__Bacteria;p__Proteobacteria;c__Alphaproteobacteria;o__Sphingomonadales;f__Sphingomonadaceae;g__Kaistobacter
	Root	6.33	0.00	k__Bacteria;p__Bacteroidetes;c__Flavobacteriia;o__Flavobacteriales;f__Flavobacteriaceae;g__Flavobacterium
		3.03	0.03	k__Bacteria;p__Proteobacteria;c__Alphaproteobacteria;o__Rhizobiales;f__Bradyrhizobiaceae; g_[Bradyrhizobium]
		2.57	0.04	k__Bacteria;p__Chloroflexi; c__Anaerolineae;o__SBR1031;f__A4b
		-4.13	0.01	k__Bacteria;p__Actinobacteria;c__Actinobacteria;o__Actinomycetales;f__Promicromonosporaceae;g__Promicromonospora
		-2.56	0.03	k__Bacteria;p__Actinobacteria;c__Actinobacteria;o__Actinomycetales;f__Actinosynnemataceae
		-1.96	0.04	k__Bacteria;p__Actinobacteria;c__Actinobacteria;o__Actinomycetales;f__Microbacteriaceae
		-2.57	0.04	k__Bacteria;p__Firmicutes;
**M+** I+ vs. M- I+	Soil	-2.97	0.01	k__Bacteria;p__Verrucomicrobia; c__Spartobacteria;o__Chthoniobacterales;f__Chthoniobacteraceae;g__DA101
		-3.40	0.01	k__Bacteria;p__Proteobacteria;c__Deltaproteobacteria;o__Syntrophobacterales;f__Syntrophobacteraceae
		-2.86	0.02	k__Bacteria;p__Nitrospirae; c__Nitrospira;o__Nitrospirales;f__0319-6A21
		-2.48	0.03	k__Bacteria;p__Proteobacteria;c__Alphaproteobacteria;o__Rhodospirillales;f__Rhodospirillaceae; g_un.
		-2.05	0.05	k__Bacteria; p__Acidobacteria; c__Chloracidobacteria; o__RB41; f_un.

Significant taxa determined by pairwise Welch's t-tests with an FDR-adjusted P value adjusted (adj-P; α = 0.05)
