# Supplementary material for: Plant microbiome analysis after Metarhizium amendment reveals increases in abundance of plant growth-promoting organisms and maintenance of disease-suppressive soil
Source: PLoS One. 2020 Apr 10;15(4):e0231150. doi: 10.1371/journal.pone.0231150 (PMC7147777; doi:10.1371/journal.pone.0231150)
Supplement: S6 Table — (PDF) [file pone.0231150.s009.pdf]

**S6 Table. Fungal taxa significantly affected by *Metarhizium robertsii* amendment determined by Welch's t-test.**

| Comparison | Location | Effect Size | adj-P | Taxon |
|---|---|---|---|---|
| **M+** I- vs. M- I- | Soil | 2.88 | 0.02 | k__Fungi;p__Ascomycota;c__Sordariomycetes;o__Hypocreales;f__Clavicipitaceae |
| | | 2.64 | 0.02 | k__Fungi;p__Chytridiomycota; c_Chytridiomycetes; o_un. |
| | | -2.04 | 0.03 | k__Fungi;p__Basidiomycota;c__Agaricomycetes;o__Agaricales;f__Hygrophoraceae;g__Hygrocybe |
| | | 2.03 | 0.04 | k__Fungi;p__Ascomycota;c__Sordariomycetes;o__Sordariales;f__Chaetomiaceae |
| | | 2.04 | 0.04 | k__Fungi;p__Chytridiomycota;c__Chytridiomycetes;o__Spizellomycetales;f__Spizellomycetaceae;g__Spizellomyces |
| | | -2.04 | 0.04 | k__Fungi;p__Ascomycota;c__Geoglossomycetes;o__Geoglossales;f__Geoglossaceae;g__Trichoglossum |
| | | -2.28 | 0.04 | k__Fungi;p__Ascomycota;c__Geoglossomycetes;o__Geoglossales;f__un. |
| | | -1.92 | 0.05 | k__Fungi;p__Basidiomycota;c__Microbotryomycetes;o__Heterogastridiales;f__Heterogastridiaceae;g__Pycnopulvinus |
| | | 2.88 | 0.02 | k__Fungi;p__Ascomycota;c__Sordariomycetes;o__Hypocreales;f__Clavicipitaceae |
| | Root | -5.50 | 0.01 | k__Fungi;p__Basidiomycota;c__Agaricomycetes;o__Russulales;f__Stephanosporaceae |
| **M+** I+ vs. M- I+ | Soil | -2.38 | 0.03 | k__Fungi;p__Ascomycota;c__Pezizomycetes;o__Pezizales;f__Ascobolaceae |
| | | -2.16 | 0.04 | k__Fungi;p__Zygomycota;c__Mucoromycotina_cls_Incertae_sedis;o__Mucorales;f__Cunninghamellaceae; g__Cunninghamella;s__]Cunninghamella_elegans] |
| | Root | -1.86 | 0.05 | k__Fungi;p__Ascomycota;c__Leotiomycetes;o__Helotiales_famIncertaesedis; f_un. |

Significant taxa determined by pairwise Welch's t-tests with an FDR-adjusted P value adjusted (adj-P; α = 0.05)
