# Supplementary material for: Plant microbiome analysis after Metarhizium amendment reveals increases in abundance of plant growth-promoting organisms and maintenance of disease-suppressive soil
Source: PLoS One. 2020 Apr 10;15(4):e0231150. doi: 10.1371/journal.pone.0231150 (PMC7147777; doi:10.1371/journal.pone.0231150)
Supplement: S7 Table — (PDF) [file pone.0231150.s010.pdf]

S7 Table. Bacterial taxa significantly affected by *Galleria mellonella* treatment determined by Welch's t-test.

Comparison	Location	Effect Size	adj-P	Taxon
**I+** M- vs. I- M-	Soil	-2.03	0.03	k__Bacteria; p__Bacteroidetes; c__Sphingobacteriia; o__Sphingobacteriales; f__Sphingobacteriaceae; g__Olivibacter
	Root	-4.16	0.01	k__Bacteria;p__Proteobacteria;c__Alphaproteobacteria;o__Ellin329; f_un.
		2.35	0.02	k__Bacteria;p__Bacteroidetes;c__Flavobacteriia;o__Flavobacteriales;f__[Weeksellaceae];g__Chryseobacterium
		-2.45	0.02	k__Bacteria;p__Bacteroidetes;c__[Saprospirae];o__[Saprospirales];f__Chitinophagaceae;g__Niastella
		-3.59	0.02	k__Bacteria;p__Proteobacteria;c__Alphaproteobacteria;o__Rhizobiales;f__Rhizobiaceae;g__Shinella
		2.85	0.03	k__Bacteria;p__Proteobacteria;c__Gammaproteobacteria;o__Legionellales;f__Coxiellaceae;g__Aquicella
		1.93	0.03	k__Bacteria;p__Firmicutes;c__Bacilli;o__Bacillales;f__Bacillaceae;g__Bacillus
		1.88	0.04	k__Bacteria;p__Verrucomicrobia;c__[Spartobacteria];o__[Chthoniobacterales];f__[Chthoniobacteraceae];g__Chthoniobacter
		2.49	0.04	k__Bacteria;p__Armatimonadetes;c__[Fimbriimonadia];o__[Fimbriimonadales];f__[Fimbriimonadaceae];g__Fimbriimonas
		2.35	0.04	k__Bacteria;p__Actinobacteria;c__Actinobacteria;o__Actinomycetales;f__Sporichthyaceae
		-2.22	0.05	k__Bacteria;p__Acidobacteria;c__[Chloracidobacteria];o__RB41
		-2.55	0.05	k__Bacteria;p__Firmicutes;c__Clostridia;o__Clostridiales;f__Ruminococcaceae
		-2.17	0.05	k__Bacteria;p__Verrucomicrobia;c__Verrucomicrobiae;o__Verrucomicrobiales;f__Verrucomicrobiaceae
**I+** M+ vs. I- M+	Soil	-0.92	0.01	k__Bacteria; p__Proteobacteria; c__Alphaproteobacteria; o__Rhizobiales; f__Bradyrhizobiaceae; g__Bradyrhizobium
		3.42	0.01	k__Bacteria; p__Actinobacteria; c__Thermoleophilia; o__Solirubrobacterales; g_un.
		2.69	0.02	k__Bacteria; p__Actinobacteria; c__Thermoleophilia; o__Gaiellales; f__Gaiellaceae; g_un.
		2.21	0.04	k__Bacteria;p__Proteobacteria;c__Alphaproteobacteria;o__Rhizobiales;f__Hyphomicrobiaceae

Significant taxa determined by pairwise Welch's t-tests with an FDR-adjusted P value adjusted (adj-P; α = 0.05)
