# Supplementary material for: Plant microbiome analysis after Metarhizium amendment reveals increases in abundance of plant growth-promoting organisms and maintenance of disease-suppressive soil
Source: PLoS One. 2020 Apr 10;15(4):e0231150. doi: 10.1371/journal.pone.0231150 (PMC7147777; doi:10.1371/journal.pone.0231150)
Supplement: S8 Table — (PDF) [file pone.0231150.s011.pdf]

S8 Table. Fungal taxa significantly affected by *Galleria mellonella* treatment determined by Welch's t-test.

Comparison	Location	Effect Size	adj-P	Taxon
**I+** M- vs. I- M-	Soil	-2.39	0.03	k__Fungi;p__Ascomycota;c__Geoglossomycetes;o__Geoglossales;f__Geoglossaceae;g__Trichoglossum
		-2.06	0.04	k__Fungi;p__Basidiomycota;c__Microbotryomycetes;o__Heterogastridiales;f__Heterogastridiaceae;g__Pycnopulvinus
		2.48	0.04	k__Fungi;p__Chytridiomycota;c__Rhizophlyctidomycetes;o__Rhizophlyctidales;f__Rhizophlyctidaceae;g__Rhizophlyctis
		2.91	0.04	k__Fungi;p__Ascomycota;c__Sordariomycetes;o__Hypocreales;f__Hypocreaceae
		-2.61	0.04	k__Fungi;p__Ascomycota;c__Eurotiomycetes;o__Chaetothyriales;f__Chaetothyriaceae
		-1.84	0.05	k__Fungi;p__Ascomycota;c__Archaeorhizomycetes;o__Archaeorhizomycetales;f__Archaeorhizomycetaceae;g__Archaeorhizomyces
	Root	2.53	0.05	k__Fungi;p__Ascomycota;c__Sordariomycetes;o__Sordariales;f__Chaetomiaceae;g__Chaetomium
		-3.05	0.01	k__Fungi;p__Basidiomycota;c__Agaricomycetes;o__Russulales;f__Stephanosporaceae
**I+** M+ vs. I- M+	Soil	1.83	0.04	k__Fungi;p__Ascomycota;c__Sordariomycetes;o__Sordariales;f__Chaetomiaceae;g__Chaetomium
		-2.06	0.05	k__Fungi;p__Kickxellomycota;c__Kickxellomycetes;o__Kickxellales; f_un

Significant taxa determined by pairwise Welch's t-tests with an FDR-adjusted P value adjusted (adj-P; α = 0.05)
