# Supplementary material for: Plant microbiome analysis after Metarhizium amendment reveals increases in abundance of plant growth-promoting organisms and maintenance of disease-suppressive soil
Source: PLoS One. 2020 Apr 10;15(4):e0231150. doi: 10.1371/journal.pone.0231150 (PMC7147777; doi:10.1371/journal.pone.0231150)
Supplement: S9 Table — (PDF) [file pone.0231150.s012.pdf]

S9 Table. Dry weight measurements for bean plants from disease-suppressive soil assay.

	Dry weight (g)		
Autoclaved soil	Root	Phyllosphere	Total
Bean	0.12 ± 0.01	0.47 ± 0.08	0.59 ± 0.09
Bean + *Fusarium*	0.12 ± 0.04	0.52 ± 0.10	0.64 ± 0.13
Bean + *Fusarium* + *Metarhizium*	0.11 ± 0.04	0.50 ± 0.11	0.61 ± 0.14
Microbiome soil	Root	Phyllosphere	Total
Bean	0.08 ± 0.02	0.35 ± 0.10	0.43 ± 0.12
Bean + *Fusarium*	0.13 ± 0.03	0.50 ± 0.06	0.63 ± 0.07
Bean + *Fusarium* + *Metarhizium*	0.11 ± 0.02	0.52 ± 0.08	0.63 ± 0.08

Plants were treated with *Fusarium solani* f. sp. *phaseoli* with or without *Metarhizium robertsii* and grown in microbiome soil or autoclaved soil for 14 days. There was no significant difference between the conditions (ANOVA, $p > 0.05$; n=3).
