# Supplementary material for: Plant microbiome analysis after Metarhizium amendment reveals increases in abundance of plant growth-promoting organisms and maintenance of disease-suppressive soil
Source: PLoS One. 2020 Apr 10;15(4):e0231150. doi: 10.1371/journal.pone.0231150 (PMC7147777; doi:10.1371/journal.pone.0231150)
Supplement: S10 Table — (PDF) [file pone.0231150.s013.pdf]

S10 Table. Proportional distribution of *Fusarium* species OTUs.

Fusarium species (match percent)	Number of OTUs	Total *Fusarium* OTUs	Percentage of total *Fusarium* OTUs
Fusarium_brasiliense(100)	17	187,666	0.009%
Fusarium_keratoplasticum(100)	3		0.002%
Fusarium_neocosmosporiellum(100)	2		0.001%
Fusarium_oxysporum(100)	184063		98.080%
Fusarium_poae(100)	1		0.001%
Fusarium_solani(100)	2429		1.294%
Fusarium_unclassified(100)	1151		0.613%
